# Increasing Photovoltaic Performance of an Organic Cationic Chromophore by Anion Exchange

**DOI:** 10.1002/advs.201700496

**Published:** 2017-12-05

**Authors:** Donatas Gesevičius, Antonia Neels, Sandra Jenatsch, Erwin Hack, Lucas Viani, Stavros Athanasopoulos, Frank Nüesch, Jakob Heier

**Affiliations:** ^1^ Laboratory for Functional Polymers Swiss Federal Laboratories for Materials Science and Technology, Empa Überlandstrasse 129 8600 Dübendorf Switzerland; ^2^ Institute of Chemical Sciences and Engineering, ISIC Ecole Polytechnique Fédérale de Lausanne, EPFL Station 6 CH‐1015 Lausanne Switzerland; ^3^ Center for X‐ray Analytics Swiss Federal Laboratories for Materials Science and Technology, Empa Überlandstrasse 129 8600 Dübendorf Switzerland; ^4^ Laboratory for Transport at Nanoscale Interfaces Swiss Federal Laboratories for Materials Science and Technology, Empa Überlandstrasse 129 8600 Dübendorf Switzerland; ^5^ Institute for Fluid Dynamics Nanoscience and Industrial Mathematics Universidad Carlos III de Madrid Avenida Universidad 30 28911 Leganés Madrid Spain; ^6^ Departamento de Física Universidad Carlos III de Madrid Avenida Universidad 30 28911 Leganés Madrid Spain; ^7^ Institut des Matériaux Ecole Polytechnique Fédérale de Lausanne, EPFL Station 6 CH‐1015 Lausanne Switzerland

**Keywords:** cyanine dyes, morphology, organic photovoltaics, single crystals

## Abstract

A symmetrical cyanine dye chromophore is modified with different counteranions to study the effect on crystal packing, polarizability, thermal stability, optical properties, light absorbing layer morphology, and organic photovoltaic (OPV) device parameters. Four sulfonate‐based anions and the bulky bistriflylimide anion are introduced to the 2‐[5‐(1,3‐dihydro‐1,3,3‐trimethyl‐2H‐indol‐2‐ylidene)‐1,3‐pentadien‐1‐yl]‐1,3,3‐trimethyl‐3H‐indolium chromophore using an Amberlyst A26 (OH^−^ form) anion exchanger. Anionic charge distribution clearly correlates with device performance, whereby an average efficiency of 2% was reached in a standard bilayer organic solar. Evidence is given that the negative charge of the anion distributed over a large number of atoms is significantly more important than the size of the organic moieties of the sulfonate charge carrying group. This provides a clear strategy for future design of more efficient cyanine dyes for OPV applications.

## 1 Introduction

Cyanine dyes are a class of organic salts, with a positively charged chromophore and a corresponding anion. A common structural leitmotif for cyanine dyes consists of a polymethine chain flanked by indolenine derivatives.^1^, ^2^, ^3^, ^4^ The first report on the synthesis of cyanine dyes dates back to 1856 and the first application of such organic salts was as color sensitizer in photography.^5^ Since then, more applications emerged in the field of life sciences as fluorescent marker reagents,^6^, ^7^ nonlinear optics,^8^, ^9^ data storage in CD‐R,^10^, ^11^, ^12^ and as organic semiconductors in organic photovoltaic (OPV) devices and photodiodes.^3^, ^13^, ^14^, ^15^, ^16^, ^17^, ^18^

Symmetrical polymethine dyes usually contain two nitrogen flanking groups. The dye in this study is an indocyanine chromophore, which consists of two symmetrical indoles connected by a polymethine chain.^19^, ^20^ Traditionally Fisher indole synthesis with a phenylhydrazine and the corresponding 3‐methyl‐2‐butanone as reactants has been used to form the half‐dye.^21^, ^22^ This can then be connected via amine condensation of a malonaldehyde bis(phenylimine) hydrochloride to a chosen polymethine chain. In modern synthesis however, microwave reactors and “more green” routes have been developed to form the half‐dyes,^23^ resulting in a higher yield, shorter reaction times, and less toxic reagents and side products.

For OPV applications, researchers have focused on tuning the highest occupied molecular orbital‐lowest unoccupied molecular orbital (HOMO–LUMO) energy gap of the chromophore. The two major strategies to do this are increasing the length of the polymethine chain by adding vinylene groups or the introduction of new terminal heterocycles.^4^

The corresponding anion of an indocyanine is defined by the reaction process, which requires methylation of the indole ring nitrogen. Because this often results in unwanted halides or halogenates (Cl^−^, I^−^, ClO_4_^−^), techniques were introduced to substitute these small and hard (Cl^−^) or partly redox active (I^−^, ClO_4_^−^) anions. At the same time new features like enhanced thermal stability and solubility were introduced.^24^ Salt metathesis using two‐phase liquid/liquid systems is the most commonly used technique.^25^, ^26^, ^27^ However, it often requires several purification steps either by recrystallization or column chromatography, which lowers the yield of the desired organic salt.

Cyanine dyes with symmetrical distribution of charge density between the two nitrogen atoms are characterized by zero bond length alternation (BLA) and show a phenomenon called the cyanine limit.^28^, ^29^, ^30^ Beyond a certain polymethine chain length, the symmetry of the electronic structure collapses. Similarly, the charge density of the chromophore can also be influenced by the corresponding anion. It was reported previously that especially hard and small anions can induce charge localization.^31^, ^32^, ^33^ Unfortunately the electronic properties of such a system cannot be predicted with the required accuracy from their delocalized analog. The anions used in this work maintain the so‐called ideal polymethine state, which is favorable for OPV applications.

Few reports have focused on the crystal structure and packing behavior of cyanine‐based organic salts.^27^, ^34^, ^35^, ^36^, ^37^ To the best of our knowledge, none of these reports has connected crystal packing with thin‐film morphology and OPV performance data. The influence of anions based on antimonates and phosphates on OPV performance in bilayer cells,^13^, ^16^ mobility, exciton diffusion length, and long‐term stability^38^ were discussed for a series of cyanine‐based organic salts. All these publications consider the near‐infrared region only, while counterion dependence of dyes absorbing in the visible region has rarely been investigated. Also, most salts require halogenated solvents for thin‐film fabrication.

In this study, we combine the products of tailored chemical engineering with the direct response from the application in OPV devices. We present a simple, general, and effective ion exchange method, which does not require halogenated solvents and was not used before in cyanine dye chemistry. The method was applied on a series of sulfonates with different organic moieties and also on a bistriflylimide anion. The size of the organic moieties bound to the negative charge carrying sulfonate group varies, while in the bulky bistriflylimide anion negative charge is distributed over several atoms. These two types of counterions were chosen to study the influence of charge distribution on electronic properties of the dye.

## 2 Results and Discussion

### 2.1 General Ion Exchange Pathway

**Figure 1:**
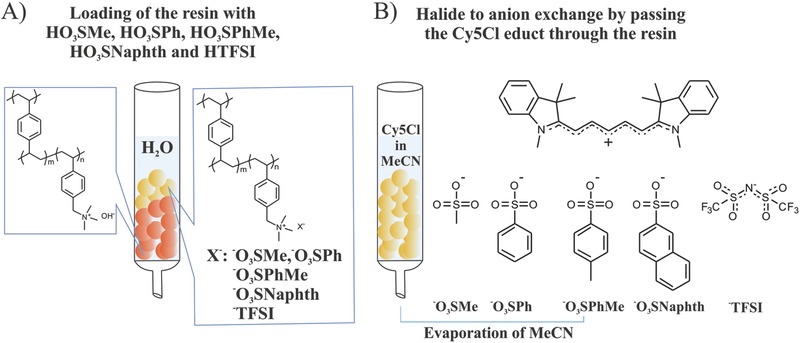
Schematic representation of the ion exchange procedure. A) The anion exchange resin is loaded with the corresponding anions. Acids are acting as anion source. B) A solution of the starting material is passed through the resin equilibrated with MeCN.

The small and hard halide anions in cyanine dye salts can have a negative influence on redox stability and charge delocalization when applied in OPV devices. To overcome this unfavorable effect, we exchanged the chloride anion in our starting material 2‐[5‐(1,3‐dihydro‐1,3,3‐trimethyl‐2H‐indol‐2‐ylidene)‐1,3‐pentadien‐1‐yl]‐1,3,3‐trimethyl‐3H‐indolium chloride by redox stable organic sulfonates or the bistriflylimide. Hereafter, we will denote these dyes as Cy5O_3_SMe, Cy5O_3_SPh, Cy5O_3_SPhMe, Cy5O_3_SNaphth, and Cy5TFSI (**Figure** **1**). The target was to introduce a one‐step ion exchange method that does not necessitate further purification steps, and that can be potentially scaled up for industry. The Amberlyst A26 (OH^−^ form; Amberlyst A26 hydroxide form is a registered trademark of The Dow Chemical Company or an affiliated company of Dow) wet anion exchange resin was chosen for this purpose (Figure 1A). Sulfonic acids and bistriflylamine are strong acids with high dissociation degree. Therefore, the corresponding anions should behave like weak bases, a desired feature for an anion. The crosslinked styrene–divinylbenzene copolymer matrix functionalized with a quaternary ammonia moiety of the anion exchange resin preferably binds small and hard anions while the larger and softer ones are released. Direct loading of the resin from aqueous solutions of the corresponding acids was achieved with a capacity of 1.2 mmol of anion per 1 g of wet anion exchange resin. The process consists of an acid‐base reaction giving water as a side product. Subsequently, after equilibration of the column with acetonitrile, salt metathesis occurs driven by the “Hard and Soft Lewis Acids and Bases” principle (Figure 1B). The ion exchange performed in this study is quantitative, and the chemically pure compounds are obtained after evaporation of all volatile compounds. The molecules were analyzed by multicore NMR spectroscopy, elemental analysis, and ion chromatography. A detailed description is provided in the ion exchange section of the Supporting Information. To the best of our knowledge this anion exchange method was applied for the first time in cyanine dye chemistry and can be expanded for a wider anion class such as carboxylates, amides, borates, nitrates, or perchlorates.

### 2.2 Anion Influence on Thermal, Optical, and Electrochemical Properties

**Figure 2:**
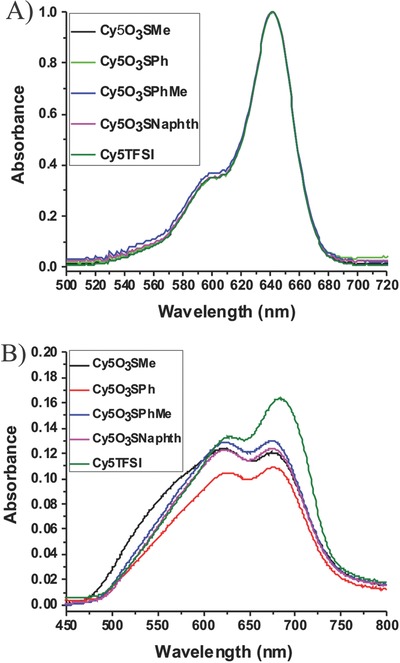
A) Normalized UV–vis spectra of the cyanine dye salts from ethanol solution. B) UV–vis spectrum of 10 nm thick films in solid state. The chromophores with sulfonate anions were spin casted from ethanol solution, while TFP was used as a solvent for the Cy5TFSI compound.

The synthesized compounds have extraordinarily high relative extinction coefficients in the range of (3.45–3.60) × 10^5^ L mol^−1^ cm^−1^. The absorption band in ethanol solution ranges from 500 to 700 nm, which is attributed to π–π* transitions (**Figure** **2**A). A strong dependence on the anion regarding the peak shape and absorbance range becomes visible in UV–vis spectra of 10 nm thick films (Figure 2B). In the solid state, the absorbance band broadens by about 4030 cm^−1^ and spans the range from 475 to 800 nm. This broadening is favorable for OPV applications and is caused to a significant extent by aggregation effects in the solid state. In ethanol solution, a vibronic shoulder shifted by 1070 cm^−1^ is visible, suggesting 0 → 0 transitions for the main peak and 0 → 1 or 0 → 2 transitions for the shoulders at shorter wavelengths caused by a dominant vibrational mode.^39^ In spincast films the shoulder is blueshifted by about 1230 cm^−1^ and reaches almost the same intensity as the absorbance maximum, pointing toward dimerization as proposed in the literature.^40^ The Cy5O_3_SMe compound shows an additional broad shoulder at shorter wavelengths, which may be a signature of H‐aggregate formation. The absorbance maximum of Cy5TFSI is redshifted by 8.1 nm compared to the absorbance maxima of chromophores with sulfonate anions, which could be caused by several synergy effects. Exciton‐band shifts, stemming from head to tail orientation of transition dipole moments of neighboring molecules, decreasing polarity of the Cy5TFSI and optical interference in thin films could lower the excitation energy. The unit cell of the corresponding crystal indeed shows such a molecular arrangement (Figure 4D). However, the HOMO–LUMO gap and the oscillator strengths obtained from ethanol solution spectra are not affected by the anion (Table S5, Supporting Information). Notable is the smaller solid state optical band gap, mainly due to the absorbance broadening toward longer wavelengths. As expected, the anion does not influence the relative HOMO and LUMO energy levels of the chromophore in dimethylformamide solution. In all compounds an electrochemical energy gap of 1.39 eV was obtained. The anions themselves are not redox active within the potential window of the chromophore or C_60_. With relative HOMO energy levels of −5.41 ± 0.01 eV and LUMO energy levels of −4.03 ± 0.01 eV, a high open circuit voltage can theoretically be expected (Table S7, Supporting Information), even though the energy levels shift in the solid state.^38^ In summary, optical absorbance, ellipsometry, and cyclic voltammetry (CV) measurements (Tables S5 and S7, Supporting Information) revealed that all synthesized organic salts are low band gap organic semiconductors, with high electron affinity and high positive oxidation potential. The observed properties render these compounds suitable candidates for donor materials in organic heterojunction solar cells. All synthesized compounds possess good relative thermal stability up to 250 °C (Figure S3, Supporting Information; Table S3, Supporting Information). Notable is the clear difference between sulfonate‐based anions and the bulkier bistriflylimide, which manifests a decomposition temperature higher by 10 °C compared to the biggest sulfonate anion. From the smallest methylsulfonate to the biggest bistriflylimide anion the decomposition resistance increases even up to 30 °C. The mass losses observed for all chromophores with sulfonate anions in the temperature range between 60 and 120 °C suggests crystal water loss, which can be reconciled with results obtained from elemental analysis. At higher temperatures an atmospheric reaction occurs with Cy5TFSI and Cy5O_3_SNaphth compounds, this mass gain could be explained by adsorption of N_2_ gas. All synthesized compounds show a two‐stage degradation, in which the integral of the first stage is of lower value than the integral of the second step. This suggests that the anion decomposes first, followed by a complex degradation of the dye backbone which occurs over a temperature range of more than 100 °C. It was not possible to identify and assign the degradation products with our thermal gravimetric analysis (TGA) setup. A clear increase of the relative decomposition temperature reflects the increasing stability of the organic salt by using anions with their negative charge distributed over several atoms.

### 2.3 Anion Influence on Crystal Packing and Electronic Structure

**Figure 3:**
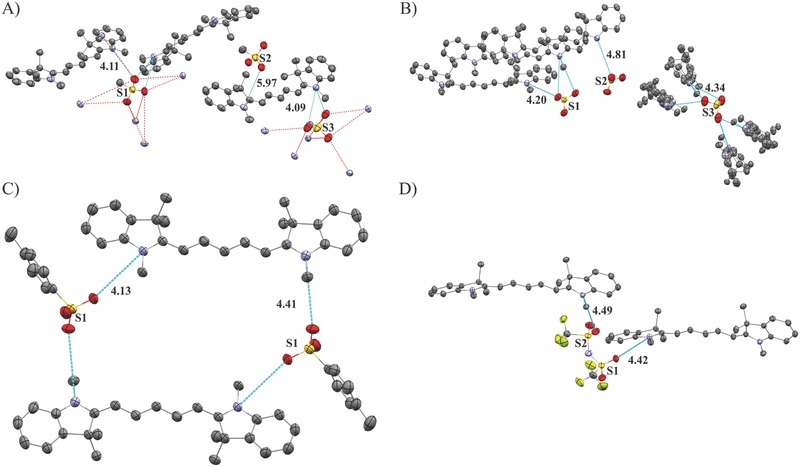
Anion coordination environment observed from single crystal data. The shortest anion–chromophore distances observed with mercury are given in Å. A) Cy5O_3_SMe, B) Cy5O_3_SPh, C) Cy5O_3_SPhMe, D) Cy5TFSI. The thermal ellipsoids represent 50% probability levels. All solvent and disordered molecules as well as hydrogen atoms are omitted for clarity. Additionally anionic contacts to the nitrogen atoms of surrounding chromophores are shown in the case of Cy5O_3_SMe and Cy5O_3_SPh. In the case of Cy5O_3_Ph only the SO_3_^−^ charge carrying group is shown for clarity. Color code: C, gray; N, violet; S, yellow; O, red; F, yellow‐green.

**Figure 4:**
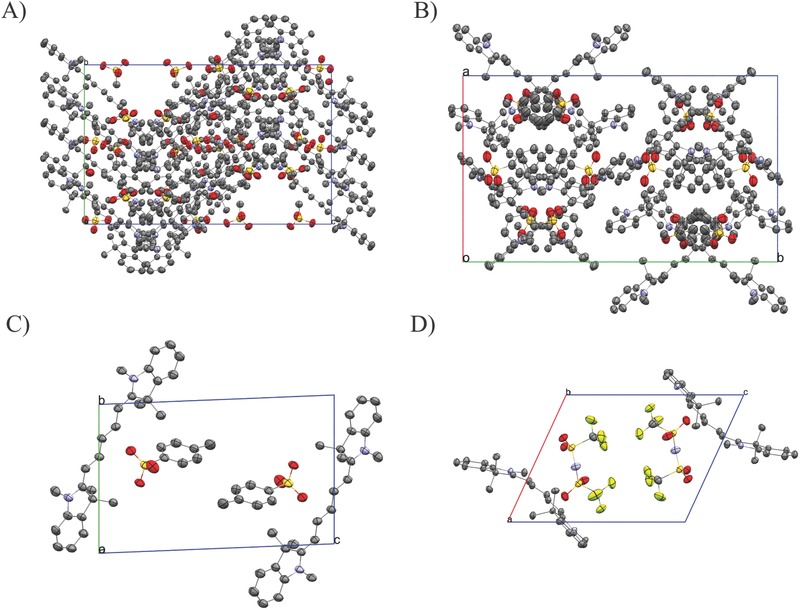
Anion influence on crystal packing, the thermal ellipsoids represent 50% probability levels. A) Cy5O_3_SMe along *a*‐axis, B) Cy5O_3_SPh packing along *c*‐axis, C) Cy5O_3_SPhMe packing along *b*‐axis, D) Cy5TFSI packing along *b*‐axis. All solvent and disordered molecules as well as hydrogen atoms are omitted for clarity. Color code: C, gray; N, violet; S, yellow; O, red; F, yellow‐green.

Single crystals suitable for X‐ray analysis could be obtained for four out of five compounds by cooling saturated solutions of the corresponding organic salts. All single crystals contained solvent molecules, and additionally, water molecules were found for Cy5O_3_SMe. Solvent, water, and disordered molecules as well as hydrogen atoms are omitted in **Figures** **3** and **4** for clarity. Symmetry lowering occurs with increasing size of the anion (**Table** **1**). A large influence of the anion on the chromophore backbone geometry can be observed. Two of the chromophores in Cy5O_3_SMe are almost planar (bending angles of 176°/175°) while one chromophore experiences a strong concave bending with an angle of 168° and additional tilt of the indolium rings with torsion angles between C13b‐C14b‐C15b‐C16b of 176.1(5)° and between C6b‐C7b‐C8b‐C9b of 177.8(5)° (Figures S13 and S14, Supporting Information; Table S11, Supporting Information). In Cy5O_3_SPh the indolium rings of two chromophores are showing an anticonformation with torsion angles between C13a/b‐C14a/b‐C15a/b‐C16a/b of 177.4(4)°/177.4(4)° and between C6a/b‐C7a/b‐C8a/b‐C9a/b of 176.5(4)°/179.5(4)°, while the third chromophore reveals an additional concave bending of 165° with a syn conformation of the nitrogen atoms. Cy5O_3_SPhMe shows an almost planar structure with torsion angles between C13‐C14‐C15‐C16 of 178.7(4)° and C6‐C7‐C8‐C9 of 177.3(3)°. The strongest concave bending of 143° and additional anticonformation of the indolium rings with torsion angles C13‐C14‐C15‐C16/C6‐C7‐C8‐C9 of 175.16(14)°/171.90(14)° occurs in Cy5TFSI. As expected, the negative charge in sulfonate based anions can be localized on one oxygen atom that has a significantly longer S–O distance, while in bistriflylimide the negative charge is delocalized over seven atoms according to the bond lengths (Table S13, Supporting Information). Therefore, we observe a clear tendency to longer chromophore–anion contacts with increasing size of the organic moiety and charge delocalization (Figure 3; Table S14, Supporting Information). In all crystal structures the anions are coordinating with the chromophore in a highly asymmetrical manner. However, a detailed insight into the coordination behavior of the anions is more complex. The anions in Cy5O_3_SMe triplets cannot be assigned to one specific chromophore. Furthermore, similar anion coordination distances indicate overlapping electrostatic interactions within the triplet (Figure 3A). In fact, each anion triplet interacts with two chromophore triplets, due to the position of the anions within two chromophore triplet layers. The chromophores are arranged in sheets separated by layers of anions. Within these sheets the chromophores form a zigzag structure with an angle of 82° (Figure 4A). All methyl sulfonate anions are coordinating asymmetrically, with a broad variation for shortest anionic contact to a nitrogen atom (4.09–5.97 Å). Neighboring chromophore molecules are stacked in staircase fashion, with shortest intermolecular carbon–nitrogen contacts between 3.57 and 3.69 Å for the indolium rings and carbon–nitrogen contacts between indolium ring and the polymethine chain of 3.62–3.64 Å as well as carbon–carbon contacts between two polymethine chains of 3.69 Å indicating weak π interactions (Figure S14, Supporting Information). Stronger π stacking is prevented by the large tilt angle of 23° within the chromophore triplet, which results in large aromatic backbone carbon–carbon distances of over 6.25 Å. A similar behavior can be found in Cy5O_3_SPh where the shortest anion contacts of the three anions lie between 4.20 and 4.80 Å (Figure 3B). The unit cell reveals a crossbone like packing, where each anion interacts with two chromophore triplets (Figure 4B). The chromophore triplets show a stacking similar to the one found in Cy5O_3_SMe, even though the tilt angles within the chromophore triplets differ. Two chromophores are facing each other with a tilt of 24° while the third chromophore faces away with a tilt of 31°. Weak intermolecular π interactions can be found between indolium rings with shortest nitrogen to carbon contacts of about 3.46 Å as well as 3.54–3.66 Å nitrogen–carbon contacts between the indolium ring and the polymethine chain (Figure S14, Supporting Information). These fluctuations in stacking angle and anion coordination distances found in Cy5O_3_SMe and Cy5O_3_SPh are also favored by solvent intercalation effects. The number of chromophore interactions per anion is reduced to two in Cy5O_3_SPhMe and Cy5TFSI (Figure 3C,D). The *p*‐methylphenylsulfonate shows 4.13 and 4.41 Å contacts to N1 and N2 of two different chromophores, while in Cy5TFSI the coordination distance increases to 4.51 and 4.49 Å for N1 and N2, respectively. The slight decrease in dielectric constant (Table S9, Supporting Information) can be correlated to the coordination distance of the anion, which reduces the polarizability. It can be assumed, that the strong geometrical distortions in the Cy5TFSI chromophore backbone are mainly caused by sterical effects of the bulky bistriflylimide anion (Figure 4D; Table S12, Supporting Information). The anion slightly influences the charge localization on the polymethine chain which can be visualized by BLA, which is here the difference between longest and shortest C—C bond length. All compounds show low BLA from 3.3 to 0.8 pm, with a general trend to lower values with increasing size and charge delocalization of the anion (Table S10, Supporting Information). The terminal nitrogen atoms show similar bond lengths between N1—C8 and N2—C14 in a range of 1.358(8)–1.361(6) Å, which is in agreement with the expected iminium fragment bond length. Notable is that the N1—C1 and N2—C21 bond lengths between 1.403(6) and 1.414(9) Å respectively, are in the range of an amine moiety, which indicates that the aromatic units of the indolium ring do not participate in the charge delocalization (Table S10, Supporting Information). Therefore, it can be assumed, that the electrons delocalized within the polymethine chain, which is terminated by the two nitrogen atoms of the indolium moieties, are forming the π‐orbitals of the chromophore. With a low maximal BLA of 0.8 pm the polymethine chain of Cy5TFSI is delocalized, despite the strongest asymmetrical coordination of the TFSI^−^ and strongest geometrical distortions of the chromophore backbone. This is in agreement with the expectation that a weakly coordinating anion should reduce interactions with the cation.

**Table 1:** Crystallographic parameters of the measured compounds. The R_1_/wR_2_ values represent the quality of the solved crystal model

Anion	^−^O_3_SMe	^−^O_3_SPh	^−^O_3_SPhMe	^−^TFSI
Crystal system	Monoclinic	Monoclinic	Triclinic	Triclinic
Space group	C2/c	P2_1_/c	P‐1	P‐1
Unit cell volume [Å^3^]	18 052	10 222	1987	1650
R_1_/wR_2_/all data	0.0880/0.2821	0.1457/0.3179	0.0607/0.1777	0.0519/0.1253

### 2.4 Anion Influence on Thin‐Film Morphology

**Figure 5:**
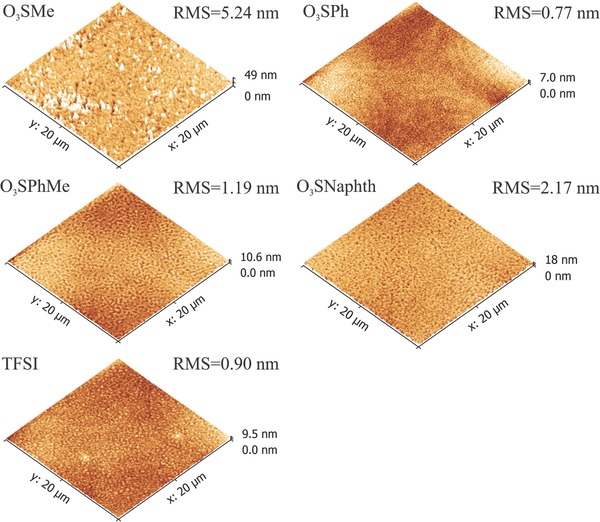
Topography of the active layer. The scanned area was fixed to 20 × 20 µm^2^ for all compounds.

To achieve uniform and defect‐free thin films of 10 nm thickness of the dye on top of the hole transporting layer is a challenging task because the cyanines tend to crystalize or dewet by forming either dye droplets or micrometer‐sized aggregates. The best cyanine layer quality was achieved from environmentally friendly ethanol solutions for the sulfonate based salts, and from 2,2,3,3‐tetrafluoro‐1‐propanol (TFP) for the bistriflylimide salt. Despite optimized spin coating conditions, the morphology of the spincast films differs significantly. The overall surface roughness parameters are low, though large differences in local surface morphology occur. The line profiles show local peaks or pinholes the number of which increases with decreasing size of the anion (**Figure** **5**). The 2‐naphthalenesulfonate anion causes a pinhole rich surface, while the Cy5O_3_SMe shows a local increase of dye layer thickness up to 40 nm. The smoothest pinhole and spike free surface is achieved with the bistriflylimide anion. Cy5O_3_SPh and Cy5O_3_SPhMe gave similar surfaces, which are slightly rougher compared to the Cy5TFSI surface. Based on AFM data, the smoothest and most defect‐free surfaces are obtained from dyes possessing the anion with larger negative charge delocalization. However, the AFM study does not allow one to draw precise conclusions of the origin of the observed thin‐film morphologies. In principle, incompatibility in surface energy between the hole transport layer and the dye in combination with residual solvent traces can cause dewetting processes. Also, dye aggregation caused by intermolecular π interactions cannot be excluded. The crystal packing diagram (Figure 4A) and the UV–vis spectrum (Figure 2B) of the chromophore with the smallest methylsulfonate anion indicate strong aggregation behavior, which could lead to locally thicker dye layers (O_3_SMe, Figure 5). The AFM measurements allow the conclusion that a larger spacing between the chromophores, which is provided by introduction of large counterions, suppresses both dewetting phenomena and aggregate formation during spin casting.

### 2.5 Anion Influence on OPV Device Performance

**Figure 6:**
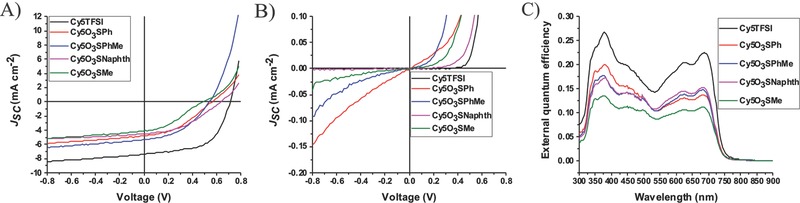
*J*–*V* and EQE curves of the best performing OPV cells. A) Cells under light illumination, B) dark current measured right after fabrication, C) external quantum efficiency of aged devices (several weeks of storage in a glovebox).

All synthesized compounds were used as light absorbing materials in a bilayer OPV device with standard geometry (Figure S15, Supporting Information). Cy5O_3_SPh, Cy5O_3_SPhMe, and Cy5O_3_SMe OPV devices show large parasitic dark currents, in the range of 0.15–0.04 mA cm^−2^ at −0.8 V (**Figure** **6**B). On the contrary, Cy5O_3_SNaphth and Cy5TFSI OPV devices show low values of 0.001 mA cm^−2^ at −0.8 V. Occurrence of low parallel resistance produces alternative current pathways within the device, which results in low *V*_OC_ and *J*_SC_ values. Especially the *V*_OC_ of Cy5O_3_SMe, Cy5O_3_SNaphth, and Cy5TFSI correlate with the roughness parameters of the thin‐film surface. A clear distinction can be made between the sulfonate anions and the bistriflylimide regarding the OPV device parameters. The organic moiety size effect of the sulfonate anions enhances the *V*_OC_, *J*_SC_, FF, and η by up to 35%, 15%, 15%, and 79%, respectively, mainly due to improved morphology without spikes and pinholes (**Table** **2**). Large improvement in all relevant OPV parameters occurs for the bistriflylimide which has negative charge delocalization over several atoms. The OPV device with Cy5TFSI gives a relative increase of *V*_OC_, *J*_SC_, FF, and η of up to 48%, 72%, 64%, and 440% compared to the Cy5O_3_SMe based device. Such a drastic increase in all relevant OPV parameters is not easy to explain by the very smooth active layer surface and large chromophore spacing alone. It can be speculated that several synergetic effects contribute substantially to the *V*_OC_ increase, like stronger stabilization of the chromophore in the excited state by the TFSI anion and a HOMO energy level shift of the chromophore in the solid state toward a larger donor–acceptor energy gap. Similar findings regarding HOMO energy levels were observed by Lunt and co‐workers performing ultraviolet photoelectron spectroscopy on cyanine dye films.^38^ Unfortunately, all devices showed short‐term degradation with a more rapid decrease of the cyanine contribution to the external quantum efficiency (EQE) than for C_60_ (Figure S20, Supporting Information). Nevertheless, the performance enhancement for Cy5TFSI devices aged for several weeks in the glovebox correlates with the EQE, which reaches its maximum at 26% for the Cy5TFSI (Figure 6C). Notable is that the TFSI anion does influence the carrier mobility. The measured values are in the range of 10^−5^ cm^2^ V^−1^ s^−1^ for high charge extraction rates which is higher than the mobilities obtained for salts with sulfonate anions (Figures S9 and S10, Supporting Information). This observation could expand the previously reported assumption that the hole mobility is mainly determined by the cation.^15^ Despite good OPV data for the best Cy5TFSI cell, the devices suffer from large variations of the measured values from cell to cell (Table 2). A remarkable improvement of reproducibility is achieved with high vacuum treatment of the Cy5TFSI active layer before device assembly, suggesting a TFP solvent residue effect (Table S16, Supporting Information). Similar findings were reported before when using TFP as solvent for spin‐coating.^41^ Remaining variations are mainly due to the multistep manual fabrication process of the devices. Further improvement trials were applied to the best Cy5TFSI compound regarding thickness adjustment, annealing at different temperatures and investigation of solvent additive effects. They are presented in Figures S16–S18 (Supporting Information). As expected, with increasing thickness of the active layer all relevant OPV parameters decrease, especially the EQE (Figure S19, Supporting Information). Such a drop can be caused by the increased resistance of the semiconducting film and exciton recombination. Therefore, 10 nm is the optimal film thickness for the Cy5TFSI, suggesting a short exciton diffusion length of ≈10 nm.

**Table 2:** Averaged OPV performance data for all investigated cells with the corresponding standard deviation of all measured cells

Anion	*N* cells	*V*_oc_ [V]	*J*_sc_ [mA cm^−2^]	FF [%]	Eff [%]
TFSI	32	0.68 ± 0.05	6.03 ± 0.49	51.53 ± 9.31	1.96 ± 0.57
O_3_SNaphth	8	0.62 ± 0.05	4.94 ± 0.35	37.84 ± 1.8	1.13 ± 0.12
O_3_SPhMe	6	0.51 ± 0.12	4.52 ± 0.42	41.5 ± 5.29	1.22 ± 0.33
O_3_SPh	8	0.51 ± 0.02	4.43 ± 0.28	35.43 ± 1.62	0.98 ± 0.07
O_3_SMe	8	0.46 ± 0.04	4.29 ± 0.16	35.05 ± 2.32	0.68 ± 0.05

## 3 Conclusion

Four sulfonate based anions with different organic moieties and the bistriflylimide anion were introduced to a 2‐[5‐(1,3‐dihydro‐1,3,3‐trimethyl‐2H‐indol‐2‐ylidene)‐1,3‐pentadien‐1‐yl]‐1,3,3‐trimethyl‐3H‐indolium chromophore to study effects that are caused either by the size of the organic moiety or charge delocalization. A two‐step halide‐for‐anion exchange method was introduced, using Amberlyst A26 (OH^−^ form) anion exchange resin, yielding the desired cyanine salts quantitatively and in high purity, without the need of halogenated solvents. Single crystal data revealed trends in anion coordination distance, influence on cyanine backbone geometry, BLA, and molecular packing. Despite asymmetrical coordination the anions maintain the ideal polymethine state which is favorable for OPV applications. The materials have good optical properties and exhibit an appropriate energy level to the acceptor material and hole transport layer for application in OPV devices. Drastic enhancement of all relevant OPV parameters was observed when the bistriflylimide anion was used with a negative charge delocalization over several atoms. The negative charge delocalization effect of the anion significantly favors the ideal polymethine state, chromophore spacing, thermal stability, redox stability, smooth surfaces of thin films, and charge carrier mobility. This initial study will allow us to design more efficient cyanine dyes for OPV applications in ongoing work.

## 4 Experimental Section

*Materials and Methods*: All chemicals and solvents were purchased from commercial sources (Aldrich, VWR, FEW Chemicals, Kurt J. Lesker) and used as received, unless otherwise stated. Reactions were carried out under air atmosphere using common lab glass ware. NMR multicore spectra were recorded on a Bruker AV‐400 spectrometer (^1^H NMR: 400 MHz, ^13^C{^1^H} NMR: 100 MHz, ^19^F{^1^H} NMR: 377 MHz). Chemical shifts (δ) are reported in ppm (parts per million) with the solvent residual signal (^1^H/^13^C{^1^H}: 7.20/79 for CDCl_3_) as reference. *J* coupling constants are given in Hz. Multiplicities are reported as singlet (s), doublet (d), triplet (t), quartet (q), and multiplet (m). Elemental analysis data were obtained from the Micro Laboratory of ETH Zürich with the instrument Leco TruSpec Micro for C, H, N, S, F, and O, while Cl was determined by ion chromatography. TGA was recorded on a Netzsch TG 209 F1.

*Device Fabrication and Characterization*: Glass/ITO substrates were cleaned in acetone (VWR, 99.5% GPR RECTAPUR), isopropanol (VWR, EMPLURA), ethanol (VWR, 99.5% AnalaR NORMAPUR), detergent (Hellmanex III, 2 wt% water solution), and finally washed four times with deionized water. Evaporation of MoO_3_ (99.97%) as the hole transport layer and C_60_ (99.5%) electron acceptor as well as tris(8‐hydroxyquinolinato)aluminum (99.99%) diffusion blocking layer and silver (99.99%) as the top electrode were performed in a glovebox using vapor deposition techniques. The pressure in the evaporation chamber did not exceed 6 × 10^−6^ mbar. The deposition rate was kept constant at 0.1 Å s^−1^. The active light absorbing layer was spin‐coated at a constant speed of 4000 rpm for 1 min in the glovebox under nitrogen atmosphere. The corresponding dye solutions (Figure S2, Supporting Information; Table S2, Supporting Information) were prepared in the glovebox under nitrogen atmosphere and were filtrated over a 0.45 µm filter before spin casting. The cell areas were defined with 3.1 and 7.1 mm^2^ by using a mask for cathode deposition. The solar cells were characterized under inert gas atmosphere on a calibrated solar simulator (Spectra Nova) using a Xe lamp with 100 mW cm^−2^ simulated AM1.5G solar irradiation. The light intensity was adjusted using a calibrated silicon reference cell from Rera Solutions. EQE was performed on a SpeQuest RR‐2100.

*Further Characterization*: UV–vis spectra were measured on a Varian Cary 50. Measurement of *n* and *k* was performed using a spectroscopic ellipsometer M2000‐VI (J.A. Woollam). Hole mobility measurement was performed on a Paios 3 instrument from Fluxim AG. The AFM analysis was performed on a scanning probe microscope Nanosurf Mobile S in tapping mode. CV measurements were performed on a PGStat 30 potentiostat (Autolab) using a three cell electrode system (Au working electrode, Pt counter electrode, and an Ag/AgCl reference electrode). Single crystal diffraction patterns were recorded on a Stoe Mark II‐Imaging Plate Diffractometer System (Stoe & Cie GmbH, Darmstadt, Germany) equipped with a graphite monochromator. Data collection was performed at −100 °C using Mo‐Kα radiation (λ = 0.71073 Å). The structures were solved by direct methods using the program SHELXS^1^ and refined by full matrix least squares on F^2^ with SHELXL^1^. The hydrogen atoms were included in calculated positions and treated as riding atoms using SHELXL‐97 default parameters. All nonhydrogen atoms were refined anisotropically. CCDC 1556701, 1556700, 1556698, 1556699 contain the supplementary crystallographic data for Cy5O_3_SMe, Cy5O_3_SPh, Cy5O_3_SPhMe, Cy5TFSI, respectively. These data can be obtained free of charge from The Cambridge Crystallographic Data Centre via http://www.ccdc.cam.ac.uk/data_request/cif. Detailed information about the background of the described experiments can be found in the Supporting Information.

## Conflict of Interest

The authors declare no conflict of interest.

## Supporting information

Supplementary
