# Supplementary material for: Increasing Photovoltaic Performance of an Organic Cationic Chromophore by Anion Exchange
Source: Adv Sci (Weinh). 2017 Dec 5;5(2):1700496. doi: 10.1002/advs.201700496 (PMC5827648; doi:10.1002/advs.201700496)
Supplement: Supplementary file 1 — Supplementary [file ADVS-5-1700496-s001.pdf]

Copyright WILEY-VCH Verlag GmbH & Co. KGaA, 69469 Weinheim, Germany, 2017.

ADVANCED SCIENCE
Open Access

Supporting Information

for *Adv. Sci.,* DOI: 10.1002/advs.201700496

Increasing Photovoltaic Performance of an Organic Cationic Chromophore by Anion Exchange

Donatas Gesevic#ius, Antonia Neels, Sandra Jenatsch, Erwin Hack, Lucas Viani, Stavros Athanasopoulos, Frank Nüesch, and Jakob Heier*

Supporting Information

Increasing Photovoltaic Performance of an Organic Cationic Chromophore by Anion Exchange

Donatas Gesevičius,[a,f] *Antonia Neels*[b]*, Sandra Jenatsch*[a]*, Erwin Hack*[c]*, Stavros Athanasopoulos*[d]*, Lucas Viani*[e]*, Frank Nüesch*[a,g] *and Jakob Heier*[a]

a. Laboratory for Functional Polymers, Swiss Federal Laboratories for Materials Science and Technology, Empa, Überlandstrasse 129, Dübendorf, Switzerland
b. Center for X-ray Analytics, Swiss Federal Laboratories for Materials Science and Technology, Empa, Überlandstrasse 129, Dübendorf, Switzerland
c. Laboratory for Transport at Nanoscale Interfaces, Swiss Federal Laboratories for Materials Science and Technology, Empa, Überlandstrasse 129, Dübendorf, Switzerland
d. Departamento de Física, Universidad Carlos III de Madrid, Avenida Universidad 30, 28911 Leganés, Madrid, Spain
e. Institute for Fluid Dynamics, Nanoscience and Industrial Mathematics, Universidad Carlos III de Madrid, Avenida Universidad 30, 28911 Leganés, Madrid, Spain
f. Institute of Chemical Sciences and Engineering, ISIC, Ecole Polytechnique Fédérale de Lausanne, EPFL, Station 6, CH-1015 Lausanne, Switzerland
g. Institut des Matériaux, Ecole Polytechnique Fédérale de Lausanne, EPFL, Station 6, CH-1015 Lausanne, Switzerland

E-mail: frank.nueesch@empa.ch

Table of Contents

General Information ... 3

General Notation for this Work ... 3

Anion Exchange Procedure ... 3

- **General Loading of the Resin** ... 3
- **Determination of Resin Capacity** ... 4
- **Synthesis of Cy5O_3SMe x H_2O** ... 4
- **Synthesis of Cy5O_3SPh** ... 5
- **Synthesis of Cy5O_3SPhMe** ... 6
- **Synthesis of Cy5O_3SNaphth x H_2O** ... 7
- **Synthesis of Cy5TFSI** ... 8

Thin Film Morphology and Thickness Evaluation ... 9

- **Screening of Suitable Solvents on Glass/MoO_3 Substrate** ... 9

Determination of Resulting Film Thicknesses After Solution Spincoating ... 9

Thermal Behaviour of the Dyes ... 11

UV-Vis absorbance ... 13

Cyclic Voltammetry for Determination of HOMO/LUMO Energy Levels ... 15

Carrier Mobility using the CELIV Method ... 19

Relative Permittivity ... 20

Organic Photovoltaic Device Fabrication ... 26

- **Statistics of the Cy5TFSI Cell** ... 27

Optimization Trials of the Cy5TFSI Cell ... 27

- **Vacuum Treatment of the Active Layer** ... 27
- **Thermal Annealing Followed After Vacuum Treatment** ... 28
- **Additive Effect** ... 29
- **Active Layer Thickness Variation** ... 30
- **Aging behavior** ... 31

DFT Calculations of Cyanine Dyes Based on Single Crystal Data ... 32

Literature ... 34

General Information

General Notation for this Work

- Dyes are Indole derivatives connected with a methine chain

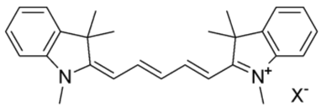

=Cy5X

2-[5-(1,3-dihydro-1,3,3-trimethyl-2H-indol-2-ylidene)-1,3-pentadien-1-yl]-1,3,3-trimethyl-3H-indolium

- Anions have sulfonates or imide as characteristic functional group

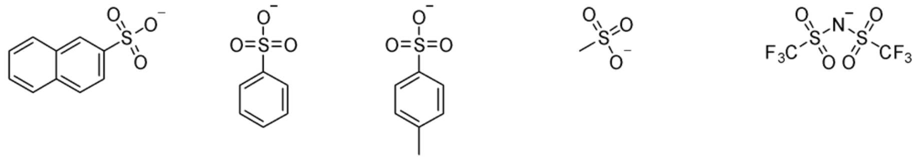

naphthalene-2-sulfonate	benzenesulfonate	*p*-methylbenzenesulfonate	methanesulfonate	bis((trifluoromethyl)sulfonyl)imide
⬇	⬇	⬇	⬇	⬇
O_3SNaphth	O_3SPh	O_3SPhMe	O_3SMe	TFSI

Figure S1. Overview of the used notation of the synthesized compounds for this work.

Anion Exchange Procedure

General Loading of the Resin

An appropriate amount of the wet anion exchange resin Amberlyst® A26 (OH^- form) was packed in a glass column (0.5 cm diameter). The column bed was then equilibrated with water until a constant pH value was reached. Then a 1% water solution of the corresponding acid was passed through the column until the eluate had reached the same pH as the original acid solution. During anion loading a change in colour of the resin from pink to pale yellow was obtained.

The pH changed from approximately 5 to 2. Then the resin was washed with water until constant pH was reached and equilibrated with the selected solvent media used for anion exchange. For the anion exchange a 1% solution of the Cy5Cl in the selected solvent mixture was passed through the column. After evaporation of the solvent the obtained residue was

dried in FV. All steps were carried out at room temperature using gravity as driving force. For the regeneration of the resin a 1 mol/L solution of NaOH was passed through the column until constant pH (12), and then washed with water again until constant pH (5).

Determination of Resin Capacity

0.1723 g (1.79 mmol) of $MeSO_3H$ 1% water solution was prepared and passed through the column. After washing of the resin with water until constant pH the amount of unspent acid was determined by volumetric titration. 0.2014 mol/L NaOH solution was prepared as titrant, 3′,3″-Dibromthymolsulfonphthaleine was used as indicator.

Table S1. Summarized data from the volumetric titration.

Compound	NaOH	$MeSO_3H$
**Starting amount (g)**	0.2014	0.1723
**Starting volume (mL)**	25	-
**Starting mole (mmol)**	5.04	1.79
**Volume titrant used (mL)**	2.8	-
**n used (mmol)**	0.564	-
**n unspent (mmol)**	-	0.564

$$C_{resin} = \frac{n_{acid} - n_{acid\ unspent}}{m_{resin}} = \frac{1.79\ mmol - 0.56\ mmol}{1\ g} = 1.2\ mmol\ g^{-1}$$

C_{resin} = capacity of the resin, n_{acid} = mole before column, $n_{acid\ unspent}$ = mole after column, m_{resin} = amount of wet anion exchange resin Amberlyst® A26 (OH^- form).

Synthesis of $Cy5O_3SMe$ x H_2O

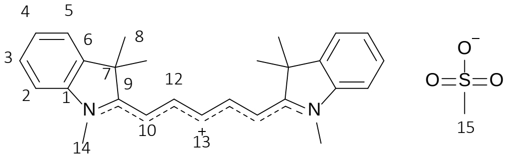

0.25 g of Cy5Cl solved in 100 mL MeCN were passed through 0.5 g of the anion exchange resin loaded with the Methanesulfonate. After evaporation of the solvent and drying for 24 h at $9.3x10^{-3}$ mbar a shiny blue solid was obtained. Yield: quantitative.

For X-Ray analysis suitable crystals were obtained by slowly cooling of a saturated chlorobenzene solution.

[$C_{28}H_{35}N_2O_4S$] 495.66 g mol^{-1}

^{1}H NMR (400 MHz, Chloroform-*d*) *δ*: 8.07 (t, *J* = 13.1 Hz, 2H, H(12)), 7.42 – 7.31 (m, 4H, H(3,4)), 7.21 (td, *J* = 7.5, 0.8 Hz, 2H, (5)), 7.11 (d, *J* = 7.9 Hz, 2H, H(2)), 6.82 (t, *J* = 12.5 Hz, 1H, H(13)), 6.33 (d, *J* = 13.7 Hz, 2H, H(10)), 3.70 (s, 6H, (H14)), 2.85 (s, 3H, H(15)), 1.71 (s, 12H, H(8)), 1.66 (s, 2H, H_2O) ppm.

^{13}C NMR (101 MHz, Chloroform-*d*) *δ*: 173.4 (C9), 153.9 (C13), 142.9 (C1), 141.0 (C6), 128.7 (C3), 125.2 (C5), 122.20 (C4), 110.6 (C2), 104.0 (C10), 49.3 (C7), 39.8 (C15), 31.9 C(14), 28.0 (C8) ppm.

Elemental analysis: Calculated: [C] 67.71, [H] 7.31, [N] 5.64, [O] 12.89, [S] 6.46.

Found: [C] 67.71, [H] 7.28, [N] 5.54, [O] 11.05, [S] 6.43.

Synthesis of Cy5O$_3$SPh

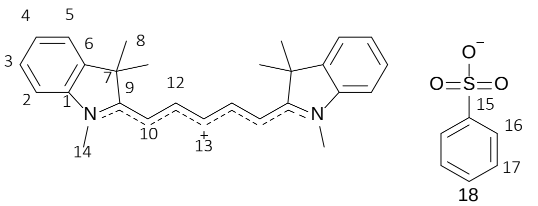

0.25 g of Cy5Cl solved in 100 mL MeCN were passed through 0.5 g of the anion exchange resin loaded with the Phenylsulfonate. After evaporation of the solvent and drying for 24 h at $9.3x10^{-3}$ mbar a shiny blue solid was obtained. Yield: quantitative.

For X-Ray analysis suitable crystals could be obtained by slow evaporation of benzene. The greenish shiny needles formed on the vial bottom.

[$C_{33}H_{35}N_2O_3S$] 539.71 g mol^{-1}

^{1}H NMR (400 MHz, Chloroform-*d*) δ: 8.22 – 7.91 (m, 4H, H(12,16)), 7.40 – 7.28 (m, 6H, H(3,4,17,18)), 7.20 (td, *J* = 7.4, 0.9 Hz, 2H, H(5)), 7.08 (d, *J* = 7.9 Hz, 2H, H(2)), 6.77 (t, *J* = 12.5 Hz, 1H, H(13)), 6.28 (d, *J* = 13.6 Hz, 2H, H(10)), 3.65 (s, 6H, H(14)), 1.66 (s, 12H, H(8)) ppm.

^{13}C NMR (101 MHz, Chloroform-*d*) δ: 173.4 (C9), 154.1 (C13), 147.8 (C15), 142.9 (C1), 141.0 (C6), 128.7 (C16), 128.7 C(17) 127.8 C(3), 126.6 (C18), 125.1 (C5), 122.2 (4), 110.5 (C2), 104.0 (C10), 49.3 (C7), 31.9 C(14), 27.9 C(8) ppm.

Elemental analysis: calculated [C] 70.94, [H] 6.85, [N] 5.01, [O] 11.45, [S] 5.74.

Found [C] 70.89, [H] 6.64, [N] 4.82, [O] 10.29, [S] 5.57.

Synthesis of Cy5O$_3$SPhMe

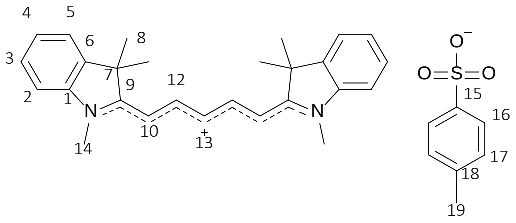

0.25 g of Cy5Cl solved in 100 mL MeCN were passed through 0.5 g of the anion exchange resin loaded with the *para* methyphenyllsulfonate. After evaporation of the solvent and drying for 24 h at 9.3x10^{-3} mbar a shiny blue solid was obtained.

For X-Ray analysis suitable crystals were obtained by slowly cooling of a saturated benzene solution.

[$C_{36}H_{43}N_2O_3S$] 583.81 g mol^{-1}

^{1}H NMR (400 MHz, Chloroform-*d*) δ: 8.09 (t, *J* = 13.1 Hz, 2H, H(12)), 7.99 – 7.82 (m, 2H, H(15)), 7.44 – 7.28 (m, 4H, H(3,4)), 7.20 (td, *J* = 7.5, 0.9 Hz, 2H, H(5)), 7.13 (d, *J* = 7.9 Hz, 2H, H(17)), 7.08 (d, 7.9 Hz, 2H, H(2)) 6.80 (t, *J* = 12.5 Hz, 1H, H(13)), 6.30 (d, *J* = 13.6 Hz, 2H, H(10)), 3.66 (s, 6H, H(14)), 2.32 (s, 3H, H(19)), 1.66 (s, 12H, H(8)) ppm.

^{13}C NMR (101 MHz, Chloroform-*d*) δ: 173.3 (C9), 154.1 (C13), 145.0 (C15), 142.9 (C1), 140.9 (C6), 138.3 (C18), 128.6 (C16), 128.3 (C17), 126.7 (C3), 125.0 (C5), 122.1 (C4), 110.5 (C2), 104.0 (C10), 49.2 (C7), 31.9 (C14), 27.9 (C8), 21.3 (C19) ppm.

Elemental analysis: calculated [C] 73.61, [H] 6.90, [N] 5.05, [O] 8.65, [S] 5.78.

Found [C] 73.49, [H] 6.94, [N] 4.84, [O] 9.04, [S] 5.34.

Synthesis of $Cy5O_3SNaphth$ x H_2O

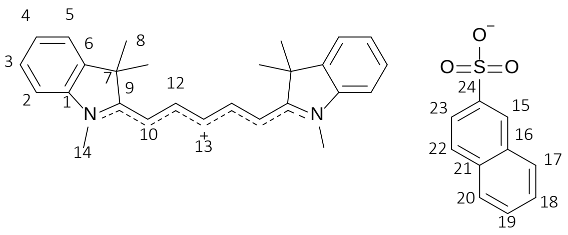

0.25 g of Cy5Cl solved in 100 mL MeCN were passed through 0.5 g of the anion exchange resin loaded with the *2*-Naphthalenesulfonate. After evaporation of the solvent and drying for 24 h at $9.3x10^{-3}$ mbar a shiny blue solid was obtained. Yield: quantitative.

[$C_{37}H_{38}N_2O_3S$] 590.78 g mol^{-1}

^{1}H NMR (400 MHz, Chloroform-*d*) δ: 8.58 – 8.50 (m, 1H, H(15)), 8.20 – 8.00 (m, 3H, H(12,23)), 7.90 – 7.82 (m, 1H, H(22)), 7.79 (dd, *J* = 7.7, 3.8 Hz, 2H, H(17,20)), 7.49 – 7.39 (m, 2H, H(19,18)), 7.38 – 7.29 (m, 4H, H(3,4)), 7.24 (d, *J* = 1.2 Hz, 2H, H(2)), 7.17 (td, *J* = 7.4, 0.9 Hz, 2H, H(5)), 7.05 (d, *J* = 7.9 Hz, 2H, H(2)), 6.74 (t, *J* = 12.4 Hz, 1H, H(13)), 6.25 (d, *J* = 13.7 Hz, 2H, H(10)), 3.61 (s, 6H, H(14)), 1.91 (s, 2H, H_2O), 1.64 (s, 12H, H(8)) ppm.

^{13}C NMR (101 MHz, Chloroform-*d*) δ: 173.4 (C9), 154.1 (C13), 145.1 (C24), 142.9 (C1), 141.0 (C6), 133.6 (C16), 132.9 (C21), 128.9 (C20,18), 127.6 (C14), 127.6 (C7), 126.2 (C19), 125.9 (C15), 125.6 (C23), 125.1 (C5) , 124.8 (C3), 122.2 (C4), 110.5 (C2), 104.0 (10), 49.3 (C7), 31.9 (C14), 27.9 (C8) ppm.

Elemental analysis: calculated [C] 73.00, [H] 6.62, [N] 4.60, [O] 10.51, [S] 5.27.

Found [C] 72.98, [H] 6.56, [N] 4.47, [O] 10.27, [S] 4.98.

Synthesis of Cy5TFSI

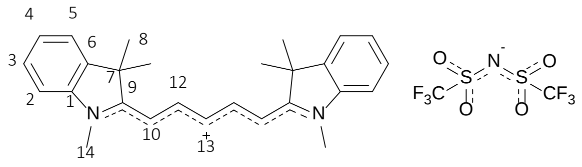

0.25 g of Cy5Cl solved in 100 mL MeCN were passed through 0.5 g of the anion exchange resin loaded with the bis(trifluoromethane)sulfonimide. After evaporation of the solvent and drying for 24 h at $1.9x10^{-2}$ mbar a shiny blue solid was obtained.

For X-Ray analysis suitable crystals were obtained by slowly cooling of a saturated ethylacetate solution.

[$C_{29}H_{31}F_6N_3O_4S_2$] 663.39 g mol^{-1}

^{1}H NMR (400 MHz, Chloroform-*d*) δ: 7.90 (t, *J* = 13.1 Hz, 2H, H(12)), 7.45 – 7.33 (m, 4H, H(3,4)), 7.25 (d, *J* = 7.5 Hz, 2H, H(5)), 7.13 (t*, *J* = 7.9 Hz, 2H, H(2)), 6.71 (t, *J* = 12.5 Hz, 1H,

H(13)), 6.24 (d, *J* = 13.6 Hz, 2H, H(10)), 3.63 (s, 6H, H(14)), 1.72 (s, 12H, H(8)) ppm. *overlap with solvent signal.

^{13}C NMR (101 MHz, Chloroform-*d*) δ: 173.4 (C9), 153.4 (C13), 142.7 (C1), 140.9 (C6), 128.7 (C3), 125.3 (C5), 122.2 (C4), 110.4 (C2), 103.8 (C10), 49.3 (C7), 31.4 (C14), 27.9 (C8) ppm.

^{19}F NMR (377 MHz, Chloroform-*d*) δ: -78.69 (s, 6F) ppm.

Elemental analysis: calculated [C] 52.48, [H] 4.71, [N] 6.33, [O] 9.64, [S] 9.66, [F] 17.17.

Found [C] 52.31, [H] 4.66, [N] 6.24, [O] -, [S] 9.72, [F] 17.20.

Thin Film Morphology and Thickness Evaluation

Screening of Suitable Solvents on Glass/MoO_3 Substrate

A 20 nm layer of MoO_3 was evaporated on glass to simulate real device conditions. All dyes were spincoated on these glass/MoO_3 substrates from preselected solvents and investigated with AFM.

Determination of Resulting Film Thicknesses After Solution Spincoating

UV-Vis spectra showed different values of absorptivity's in processed films despite that the initial dye concentration was kept constant. Obviously the usage of different solvents produces different films thicknesses. This makes tuning of individual film thicknesses necessary for each dye-solvent system. Ellipsometry was chosen as a measurement technique **(Figure S11, S12**). The used substrate is a 1 mm thick microscopic glass coated with a 20 nm MoO_3 layer. The aim is to find the right concentration for each dye that gives 10 nm thick films on MoO_3 layers. For all dyes the chosen spin coating speed was 4000 rpm for 60 sec.

Table S2. Thickness-concentration dependence for the compounds. *Determined only from that concentration.

c (mol L^{-1})	M_{dye} (g mol^{-1})	m_{dye} (mg)	$V_{Solv.}$ (mL)	d_{film} (nm)	$n_{\lambda(683\ nm)}$	$k_{\lambda(683\ nm)}$
			**$Cy5O_3SPh*(H_2O)$ from Ethanol**			
**$1*10^{-3}$**	558.74	0.4	0.7	4.0		
**$5*10^{-3}$**	558.74	2.0	0.7	13.9		
**$7.38*10^{-3}$**	558.74	2.9	0.7	24.4		
**$1*10^{-2}$**	558.74	3.9	0.7	40.0	2.57^*	1.11^*
			**$Cy5O_3SPhMe$ from Ethanol**			
**$1*10^{-3}$**	554.75	0.4	0.7	6.0		
**$5*10^{-3}$**	554.75	1.9	0.7	15.4		
**$7.38*10^{-3}$**	554.75	2.9	0.7	21.1		
**$1*10^{-2}$**	554.75	3.9	0.7	29.3	2.57^*	1.13^*
			**$Cy5O_3SNaphth*(H_2O)$ from Ethanol**			
**$1*10^{-3}$**	608.80	0.4	0.7	4.1		
**$5*10^{-3}$**	608.80	2.1	0.7	18.5		
**$7.38*10^{-3}$**	608.80	3.2	0.7	20.3		
**$1*10^{-2}$**	608.80	4.3	0.7	33.3	2.56^*	1.17^*
			**$Cy5O_3SMe*(H_2O)$ from Ethanol**			
**$1*10^{-3}$**	496.67	0.4	0.7	5.3		
**$5*10^{-3}$**	496.67	1.7	0.7	13.1		
**$7.38*10^{-3}$**	496.67	2.6	0.7	19.6		
**$1*10^{-2}$**	496.67	3.5	0.7	26.0	2.58^*	1.01^*
			**Cy5TFSI from TFP**			
**$1*10^{-3}$**	663.69	0.5	0.7	1.4		
**$5*10^{-3}$**	663.69	2.3	0.7	9.7		
**$7.38*10^{-3}$**	663.69	3.4	0.7	14.4		
**$1*10^{-2}$**	663.69	4.7	0.7	18.7	2.54^*	1.24^*

The obtained thickness values were plotted against concentration. With the linear fit equation it is possible to determine the concentration of a dye needed for a certain film thickness on a glass/MoO_3 substrate.

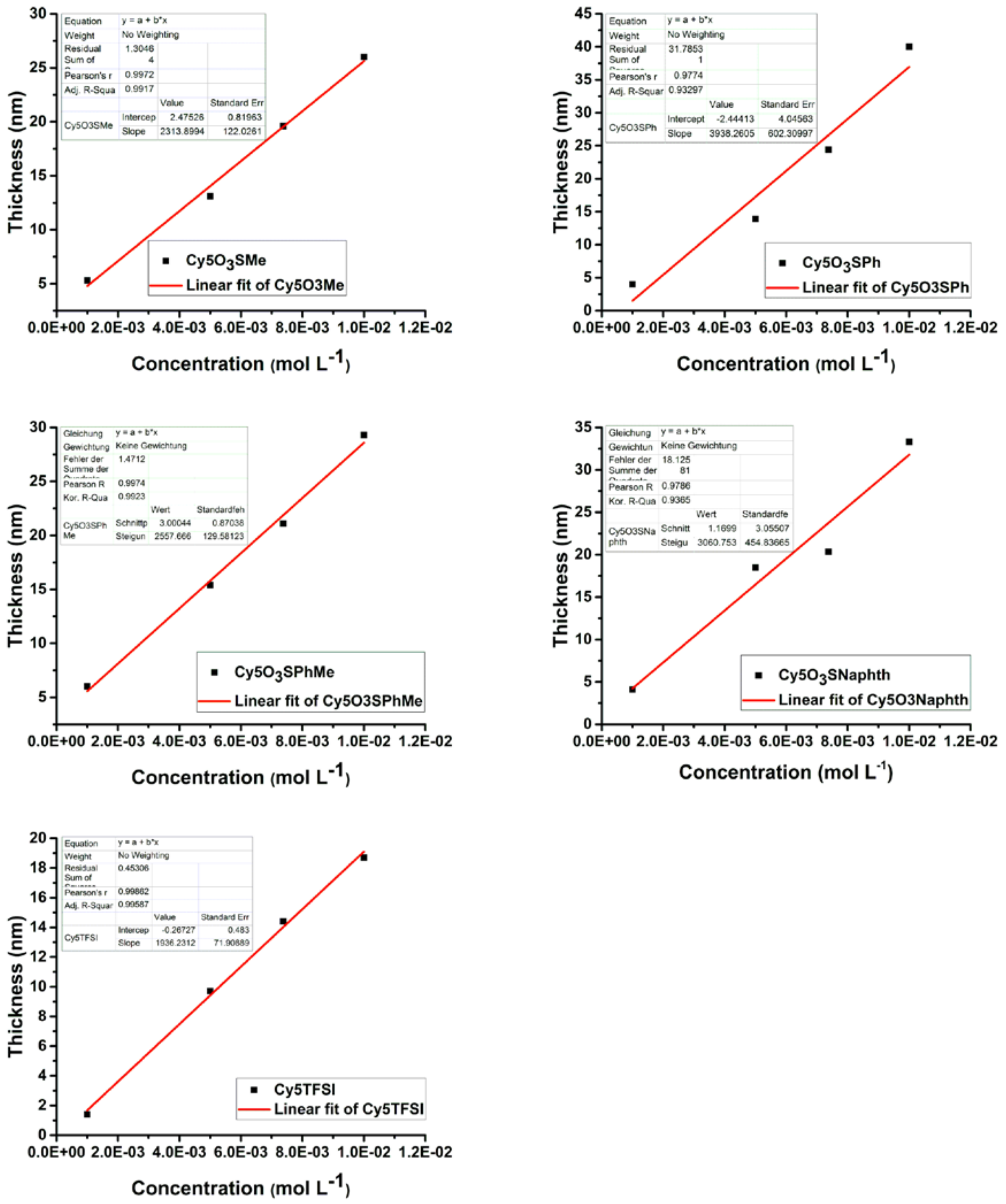

Figure S2. Thickness as a function of concentration for the used cyanines on glass/MoO_3 substrate.

Thermal Behaviour of the Dyes

The samples were weighed under air atmosphere, while the measurement was performed under nitrogen flow in a temperature range from 20-600 °C. From 600-900 °C the sample was exposed to oxygen.

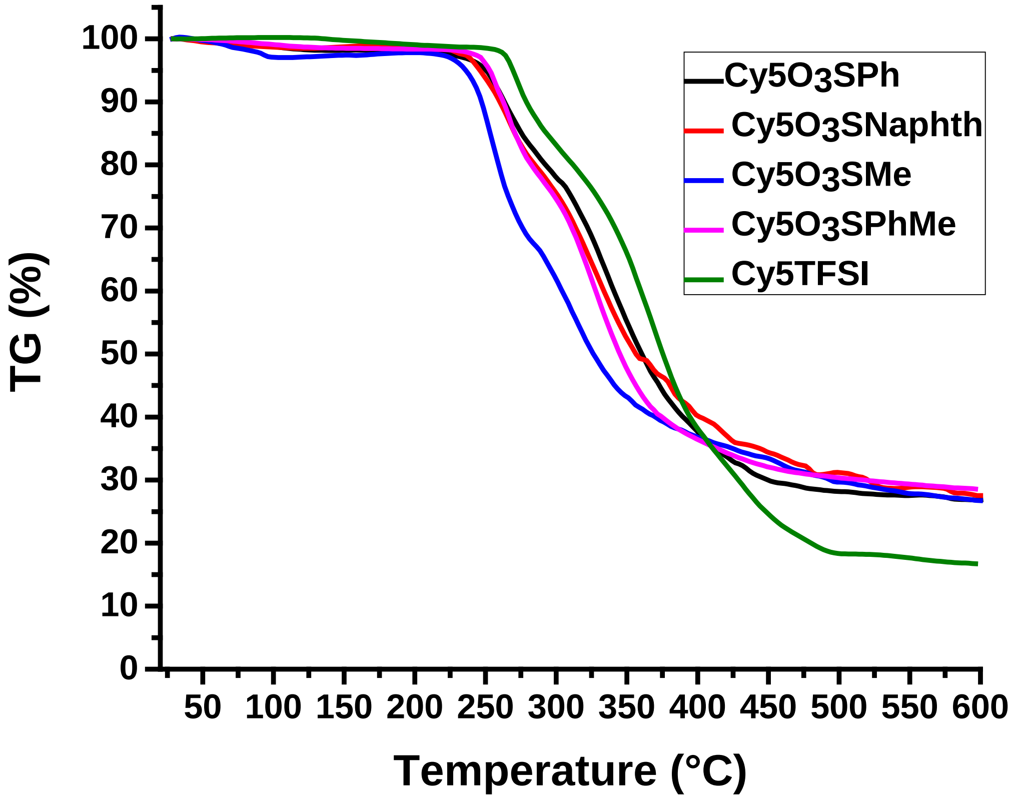

Figure S3. TGA curves of the compounds.

Table S3. Decomposition temperatures and calculated integrals from the TGA data.

**Cyanine**	**Decomp. Temp/°C**	**First step (Mid-point)**	**First step Integral**	**Second step (Mid-point)**	**Second step Integral**
**$Cy5O_3SPh$ *(H_2O)**	235	235-302 (263)	21	302-600 (337)	51
**$Cy5O_3SPhMe$**	250	250-286 (267)	21	286-600 (329)	50
**$Cy5O_3SNaphth$*(H_2O)**	240	240-280 (261)	18	280-600 (310)	43
**$Cy5O_3SMe$*(H_2O)**	230	230-283 (253)	30	283-600 (310)	41
**Cy5TFSI**	260	260-305 (271)	19	305-600 (370)	65

UV-Vis absorbance

To determine the molar extinction coefficient ethanolic solutions of the cyanine compounds of 6.37 x 10^{-4} mol/L each were prepared. Subsequently solutions with three different concentrations were prepared by diluting the initial solution (**Table S4**).

Table S4. Used concentrations for the generation of calibration points in UV-Vis.

Solution	Compound concentration c (mol L^{-1})				
	**Cy5O$_3$SMe *(H$_2$O)**	**Cy5O$_3$SPh *(H$_2$O)**	**Cy5O$_3$SPhMe**	**Cy5O$_3$SNaphth *(H$_2$O)**	**Cy5TFSI**
**1**	3.469x10^{-7}	4.550x10^{-7}	7.341x10^{-7}	6.184x10^{-7}	4.927x10^{-7}
**2**	1.363x10^{-6}	1.539x10^{-6}	1.783x10^{-6}	1.262x10^{-6}	9.352x10^{-6}
**3**	2.393x10^{-6}	3.509x10^{-6}	4.123x10^{-6}	3.168x10^{-6}	2.363x10^{-6}

All measurements were performed in a 0.1 mm quartz glass cuvette using 99.8 % ethanol as reference for the baseline. The relative molar extinction coefficient for each compound was calculated by dividing the slope of the resulting plots of concentration against absorbance intensity by 10^{-1} cm.

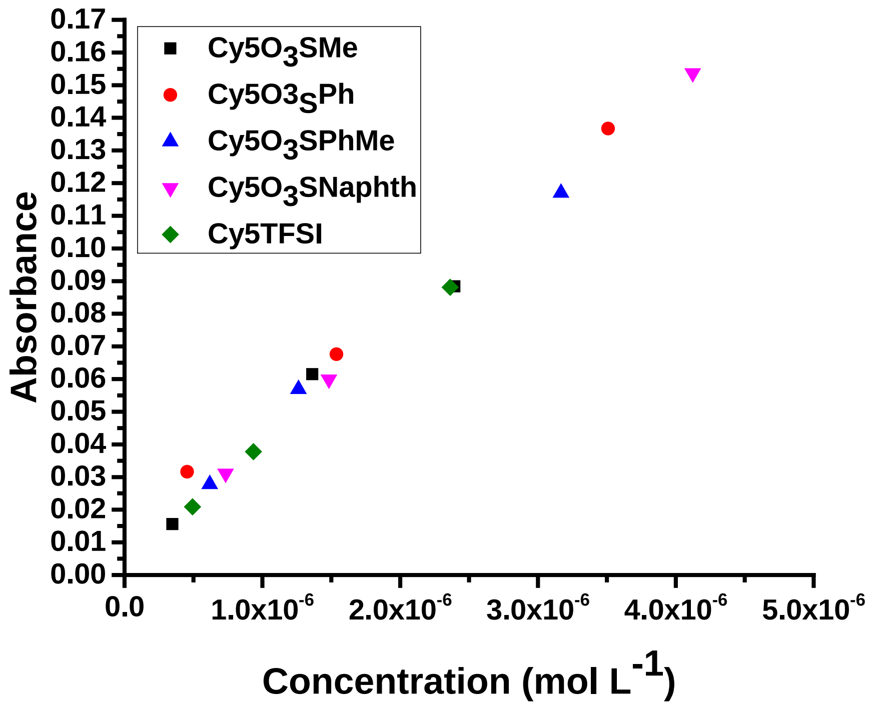

Figure S4. Concentration dependent absorbance.

The calculated extinction coefficients are summarized in **Table S5**. Since the cyanine dyes are known for the large exciton binding energy which results in strong bounded electron-hole

pairs it, becomes clear that the optical bandgap and the electronic bandgap energies differ. A photon can have enough energy to create an exciton but not enough to separate the exciton into free electron and hole, so that the optical bandgap energy is much lower than the electrical band gap energy. The optical band gap can be calculated from the onset of the absorbance at higher wavelengths with the following equation.

$$E_{g(opt)} = \frac{h \times c}{\lambda_{onset}}$$

λ_{onset}: Onset of absorption band at higher wavelength, h: Planck constant, c: speed of light.

One further value helpful to predict the excited state behaviour is the oscillator strength. The oscillator strength describes the probability of a transition from a lower to an upper energy state. The higher the value the easier the electrons can be excited and the stronger absorbing is the dye.

$$f = 4.319 \times 10^{-9} \int \varepsilon(\upsilon)\, d\upsilon$$

$\varepsilon(\upsilon)$: Molar extinction coefficient as a function of wavenumber, υ: Wavenumber.

First the wavelength was converted into wavenumbers with the following formula:

$$\upsilon = 1/(\lambda * 10^{-7})$$

Then the extinction coefficient was calculated for each wavenumber with the following formula:

$$\varepsilon_{(\upsilon)} = A/(c * d)$$

c: concentration in mol·L^{-1}, d: thickness of cuvette in cm, A: absorbance.

The calculations were performed for each recorded data point of the spectra. The resulting values were plotted, the wavenumber scale is reversed.

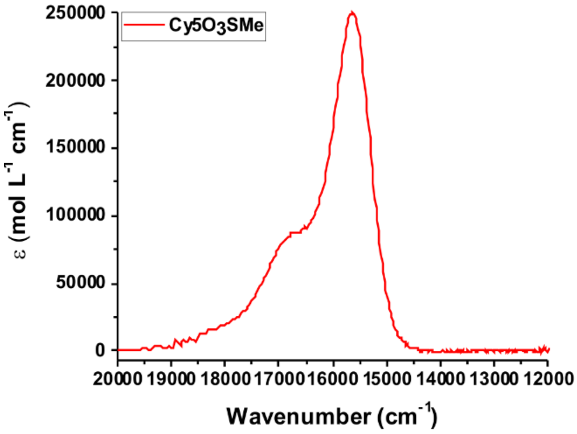

Figure S5. Extinction coefficient ε as function of wavenumber for Cy5O_3SMe solution.

The peak in **Figure S5** was assumed to represent the full band of the lowest energy π-π^* transition and was integrated to calculate the oscillator force.

Table S5. Calculated data from recorded UV-Vis spectra. * Onset energy at higher wavelengths obtained from EtOH solution, ** obtained from thin film.

Compound	ε_{max} (L mol^{-1} cm^{-1})	λ_{max} (nm)	λ_{onset} (nm)	$E_{(onset)}$* (eV)	f	$E_{g(opt)}$** (eV)
**Cy5O_3SPh* (H_2O)**	3.56 x10^5	641	672	1.74	1.51	1.68
**Cy5O_3SPhMe**	3.45 x10^5	641	672	1.74	1.63	1.68
**Cy5O_3Naphth *(H_2O)**	3.60 x10^5	641	674	1.74	1.10	1.68
**Cy5O_3SMe* (H_2O)**	3.56 x10^5	641	675	1.74	1.10	1.68
**Cy5TFSI**	3.58 x10^5	641	670	1.75	1.59	1.67

Cyclic Voltammetry for Determination of HOMO/LUMO Energy Levels

Cyclic voltammetry (CV) measurements were performed on a PGStat 30 potentiostat (Autolab) using a three cell electrode system (Au working electrode, Pt counter electrode and an Ag/AgCl reference electrode). Two electrolyte solutions of tetrabutylammonium perchlorate and tetrabutylammonium chloride were prepared in DMF each 0.1 mol/L. Each measurement needs 50 ml of tetrabutylammonium perchlorate (25 mL account for the

measurement and 25 mL for cleaning) and 10 mL of tetrabutylammonium chloride (4 mL for measurement and 6 mL for cleaning) solutions. Following amounts of the dyes were used (**Table S6**).

Table S6. Weighted quantities of the dyes.

Dye	m (mg)	n (mol)
**$Cy5O_3SPh*(H_2O)$**	10.2	$1.82x10^{-5}$
**$Cy5O_3SPhMe$**	5.1	$9.19x10^{-6}$
**$Cy5O_3SNaphth*(H_2O)$**	10.2	$1.68x10^{-5}$
**$Cy5O_3SMe*(H_2O)$**	10.1	$2.03x10^{-5}$
**Cy5TFSI**	9.6	$1.45x10^{-5}$

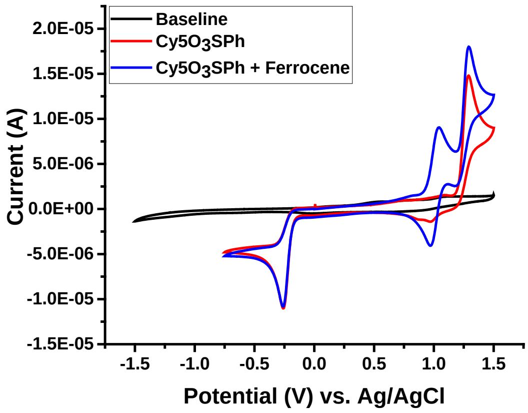

Figure S6. Recorded cyclic voltammetry data for the Cy5O3SPh dye.

The Ferrocene solution was prepared qualitatively by adding a spatula of ferrocene in 10 mL of electrolyte solution. All potentials were referenced to NHE by adopting a potential of +0.72 V vs. NHE for Fc/Fc+ in DMF.[3] The rotating disk was equilibrated before first measurement for 30 min at 3000 rpm. Then the rotation speed was reduced to 50 rpm and was kept constant for all measurements. Before each measurement step the solution was fumigated with argon for 15 min. The solvent window was determined by running 30 cycles from -1.5 V until 1.5 V with a scanning rate of 2 V/s, subsequently the scanning rate was reduced to 0.1 V/s and the baseline curve was recorded. Then the corresponding dye was added to the solution and the above described measurement procedure was repeated. The negative potential window was adjusted to -0.75 V for all chromophores.

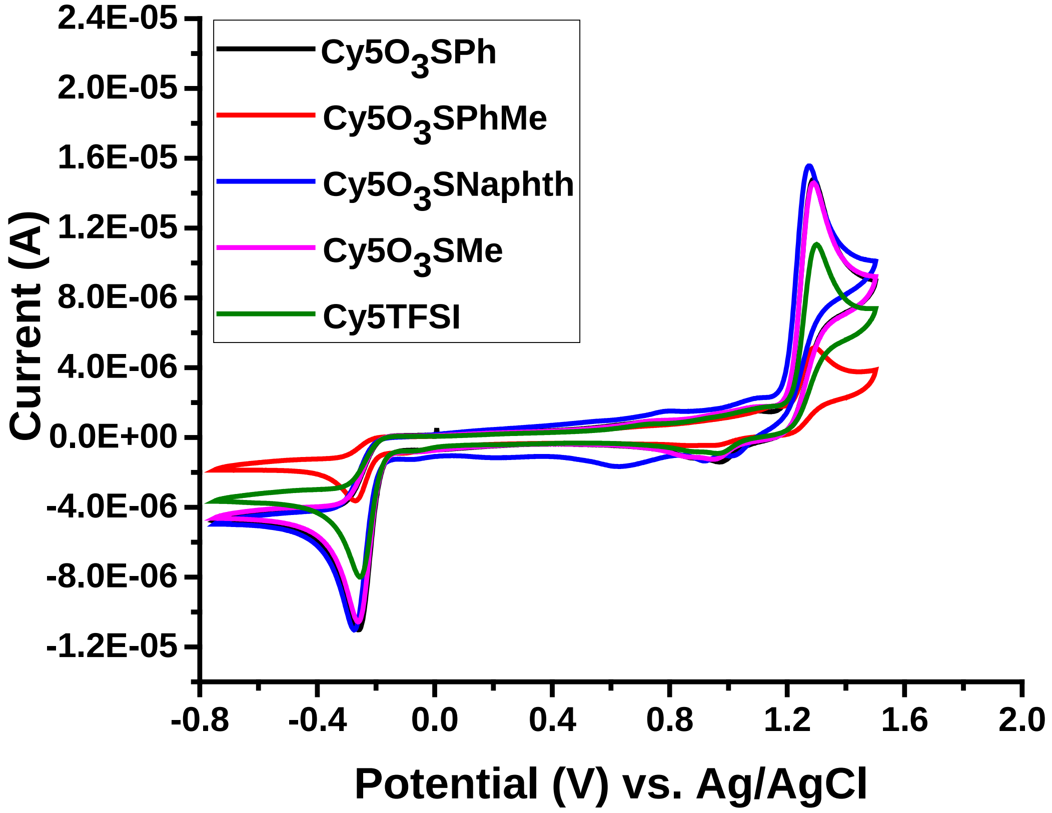

Figure S7. Cyclic voltammograms of all compounds.

The calculations of the $E_{g(el)}$ (HOMO/LUMO gap) are based on the assumption that a positive cathodic current can be referred to a reduction process, while a negative anodic current to an oxidation process. Therefore the oxidation potential corresponds to electron extraction from the HOMO level, while the reduction potential is associated with the electron affinity and indicates the LUMO level. By analysing the recorded spectra graphically it is possible to determine the respective reduction $E^{onset}_{red(dye)}$ or oxidation $E^{onset}_{ox(dye)}$ onset potentials. It is notable that the recorded cyclic voltammograms are showing irreversible processes for all investigated dyes, so that the intersection onset of the corresponding peak has to be chosen as the respective potential. While for the ferrocene which undergoes a reversible process the potential is calculated according to:

$$E^{ox}_{1/2(Ferrocene)} = \frac{E^{ox}_{pc} + E^{ox}_{pa}}{2}$$

$E^{ox}_{1/2(Ferrocene)}$: half-curve potential, E^{ox}_{pa}: anodic peak potential, E^{ox}_{pc} : cathodic peak potential.

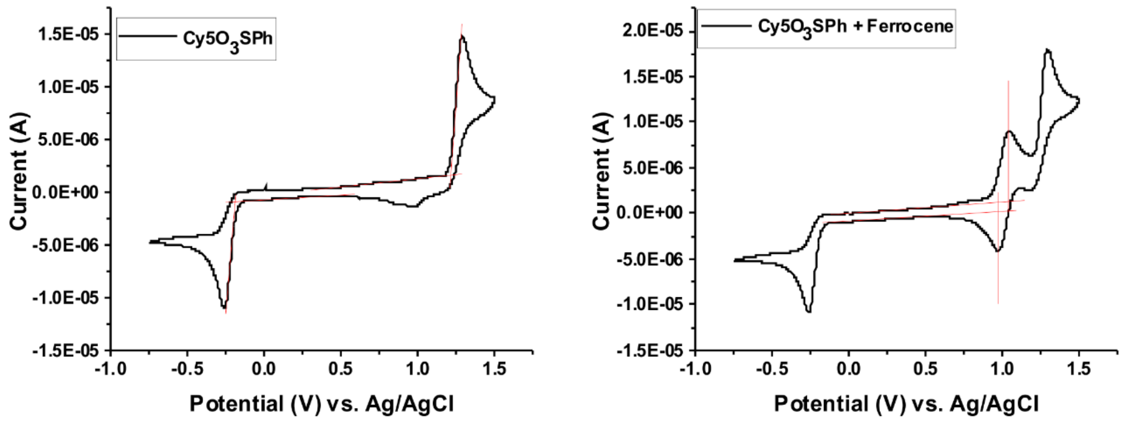

Figure S8. Drawn lines as example for intersection determination in $Cy5O_3SPh$ (left) for the dye and $Cy5O_3SPh$ + Ferrocene (right) for the Ferrocene reference.

The potentials were measured against a Ag/AgCl reference. The used conversion constant for ferrocene in DMF is 0.72 V.[3] The correction value against NHE for ferrocene was calculated as follows:

$$\text{Korr.}_{\text{Ferrocene}} = 0.72 - E^{ox}_{1/2(Ferrocene)}$$

To calculate the corrected values for the onset potentials against NHE potential the following assumptions were made:

$$E^{onset}_{ox(dye)\ vs.\ NHE} = E^{onset}_{ox(dye)} + Korr._{Ferrocene}$$
$$E^{onset}_{red(dye)\ vs.\ NHE} = E^{onset}_{red(dye)} + Korr._{Ferrocene}$$

The calculation of the HOMO and LUMO was performed by using empirical equations.[4] The used onset potentials were corrected against NHE as described above.

$$E_{HOMO} = -\left(E^{onset}_{ox(dye)\ vs.\ NHE} + 4.5\right)eV$$
$$E_{LUMO} = -\left(E^{onset}_{red(dye)\ vs.\ NHE} + 4.5\right)eV$$
$$E_{g(el)} = E_{HOMO} - E_{LUMO}$$

Table S7. Calculated data from the CV measurement.

Substance	$E^{onset}_{ox(dye)}$ /V	$E^{onset}_{red(dye)}$/V	$E^{0}_{\frac{1}{2}ox(FeCp_2)}$/V	E_{HOMO}/eV	E_{LUMO}/eV	$E_{g(el)}$/eV
**$Cy5O_3SPh$**	1.20	-0.19	1.01	-5.41	-4.02	1.39
**$Cy5O_3SPhMe$**	1.20	-0.19	1.00	-5.42	-4.03	1.39
**$Cy5O_3SNaphth$**	1.18	-0.19	1.00	-5.40	-4.03	1.38
**$Cy5O_3SMe$**	1.20	-0.18	1.00	-5.42	-4.04	1.38
**Cy5TFSI**	1.21	-0.17	1.01	-5.42	-4.04	1.38

Carrier Mobility using the CELIV Method

$$\mu = \frac{2d_{dye}\left[d_{dye} + \left(\frac{\varepsilon_{dye}}{\varepsilon_{C_{60}}}\right) \times d_{C_{60}}\right]}{3At^2{}_{max}} \times \frac{1}{\left[1 + 0.36\left(\frac{\Delta j}{j_0}\right)\right]}$$

t_{max} : time at maximum current, d_{dye} : thickness of the dye film, d_{C60} : thickness of the C_{60} layer, $\varepsilon_{dye/C_{60}}$: dielectric constants of the dye and C_{60}, Δj: peak height, j_0 : capacitive current, A : slope of the voltage.

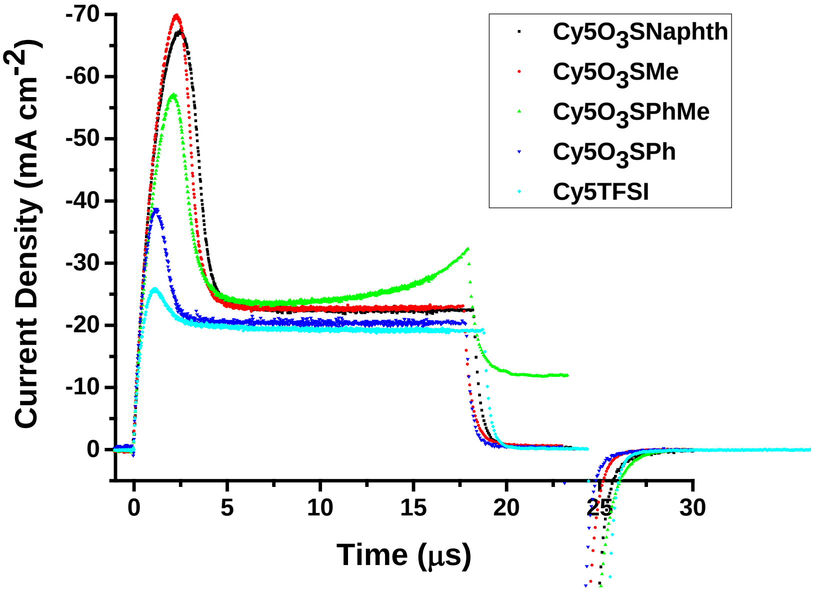

Figure S9. Time dependent current density.

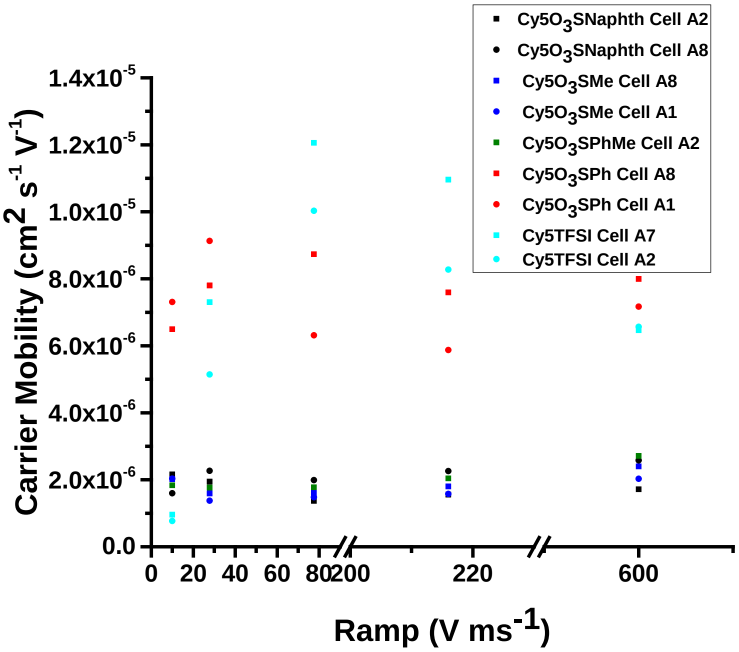

Figure S10. Carrier mobility vs the voltage ramp.

Table S8. Calculated mobilities for the cyanine compounds at a ramp of 216 V/ms. T_{max}/τ_{RC} acts as an indicator for the reliability of the values.

Cyanine	T_{max}/τ_{RC}	μ ($cm^2\ V^{-1}\ s^{-1}$)
**$Cy5O_3SPh*(H_2O)$**	6.20	$7.59x10^{-6}$
**$Cy5O_3SPhMe$**	10.77	$2.04x10^{-6}$
**$Cy5O_3SNaphth*(H_2O)$**	17.86	$2.26x10^{-6}$
**$Cy5O_3SMe*(H_2O)$**	17.07	$1.79x10^{-6}$
**Cy5TFSI**	11.09	$1.10x10^{-5}$

Relative Permittivity

$$\varepsilon_r = n^2 - k^2$$

ε_r: relative permittivity (dielectric constant or function), n: real part of the index of refraction, k. imaginary part of the index (extinction coefficient)

The orientational polarization or dipole polarisation appears at low frequencies around 10^4 Hz. The n and k values are dependent on the wavelengths. At higher wavelengths however the slope is very low and at a certain wavelength the k value becomes 0.

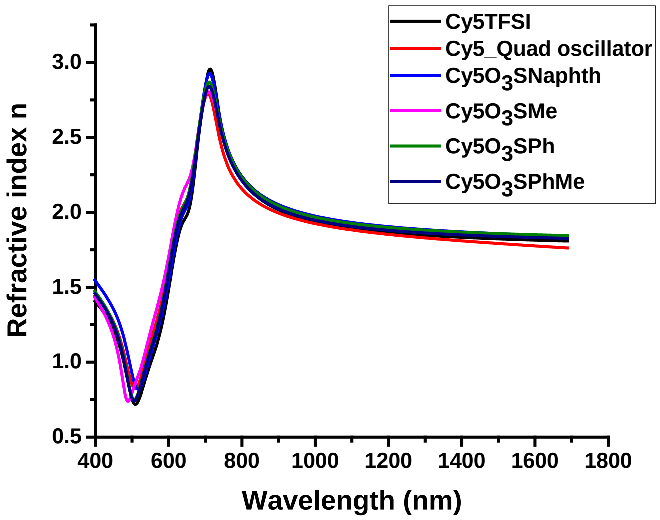

Figure S11. Function of n depending on the wavelength.

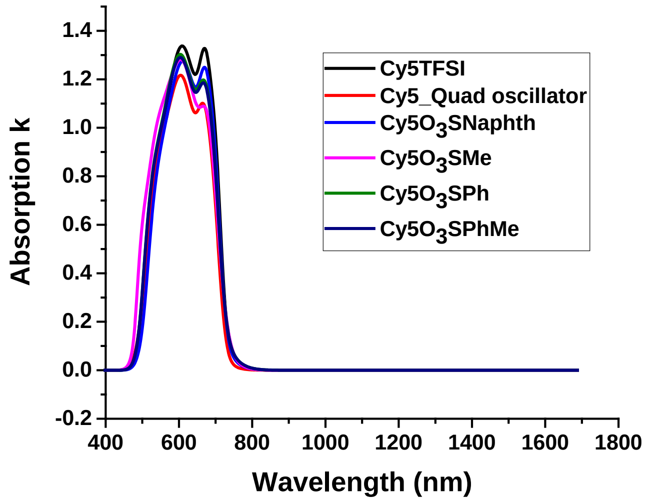

Figure S12 Function of k depending on the wavelength.

Therefore this area was chosen for the calculation of the dielectric constant. The n are given in this table as averaged values over the selected wavelength region.

Table S9. Calculated relative permittivity values for the cyanines.

Cyanine	n	λ/nm	ε_r
**Cy5O$_3$SPh*(H$_2$O)**	1.88	979-1688	3.53
**Cy5O$_3$SPhMe**	1.88	975-1688	3.53
**Cy5O$_3$SNaphth*(H$_2$O)**	1.89	965-1688	3.57
**Cy5O$_3$SMe*(H$_2$O)**	1.88	1010-1688	3.53
**Cy5TFSI**	1.86	973-1688	3.46

Crystal Structures

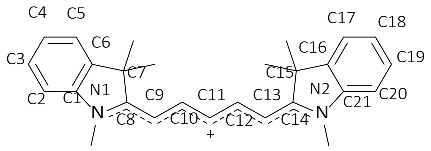

Figure S13. Cyanine atom numbering.

The cyanines were numbered according to the cif file. In the case of Cy5O_3SMe and Cy5O_3SPh compounds with chromophore/anion triplets in the asymmetric unit the chromophores are numbered with additional b/c letters. This deviation from the cif file is necessary for clarity. The relevant anion atoms were numbered according to the cif file. In the case of Cy5O_3SMe and Cy5O_3SPh with anion triplets in the asymmetric unit the anions are described with the central S atom as S1/S2/S3.

Table S10. Anion influence on bond lengths (Å) of the chromophore polymethine chain. *Difference between longest and shortest bond length.

Atom	Anion			
	O_3SMe	O_3SPh	O_3SPhMe	TFSI
**N1-C1**	1.405(6)	1.403(6)	1.408(4)	1.4089(16)
**N1b-C1b**	1.406(9)	1.415(6)		
**N1c-C1c**	1.412(6)	1.409(6)		
**N1-C8**	1.358(8)	1.356(5)	1.357(4)	1.3560(16)
**N1b-C8b**	1.363(6)	1.364(5)		
**N1c-C8c**	1.344(9)	1.362(5)		
**C8-C9**	1.376(7)	1.392(6)	1.377(5)	1.3850(18)
**C8b-C9b**	1.384(9)	1.388(6)		
**C8c-C9c**	1.395(7)	1.371(6)		
**C9-C10**	1.398(9)	1.378(6)	1.380(5)	1.3939(19)
**C9b-C10b**	1.392(7)	1.396(6)		
**C9c-C10c**	1.363(7)	1.400(6)		
**C10-C11**	1.378(6)	1.384(6)	1.388(5)	1.3874(19)
**C10b-C11b**	1.373(9)	1.386(6)		
**C10c-C11c**	1.389(7)	1.367(6)		
**C11-C12**	1.396(10)	1.387(6)	1.382(5)	1.3878(19)
**C11b-C12b**	1.390(7)	1.393(6)		

Atom	Anion			
	O_3SMe	O_3SPh	O_3SPhMe	TFSI
**C11c-C12c**	1.372(10)	1.399(6)		
**C12-C13**	1.391(7)	1.395(6)	1.391(5)	1.3896(19)
**C12b-C13b**	1.375(9)	1.398(6)		
**C12c-C13c**	1.395(7)	1.382(6)		
**C13-C14**	1.379(10)	1.385(6)	1.378(5)	1.3907(19)
**C13b-C14b**	1.398(7)	1.386(6)		
**C13c-C14c**	1.379(10)	1.387(6)		
**C14-N2**	1.361(6)	1.353(5)	1.350(4)	1.3510(17)
**C14b-N2b**	1.349(8)	1.368(5)		
**C14c-N2c**	1.360(6)	1.347(5)		
**N2-C21**	1.414(9)	1.403(6)	1.414(4)	1.4084(17)
**N2b-C21b**	1.409(6)	1.419(5)		
**N2c-C21c**	1.416(9)	1.418(5)		
**BLA***	2.2	1.7	1.3	0.8
	2.5	1.2		
	3.2	3.3		

Table S11. Anion influence on bending of the chromophore skeleton and indolenium ring conformation. Plane angle measurements between two indolenium rings were performed with Mercury 3.8 (°).

Atoms	Anions			
	O_3SMe	O_3SPh	O_3SPhMe	TFSI
**Plane angle between two indolenium rings (N conformation relatively to each other)**	176.48 (planar)<br>168.06 (syn)<br>175.63 (planar)	169.11 (anti)<br>169.65 (anti)<br>164.85 (anti)	176.32 (planar)	143.42 (anti)
**C13-C14-C15C-16**	179.2(5)	177.4(4)	178.7(4)	175.16(14)

Atoms	Anions			
	O_3SMe	O_3SPh	O_3SPhMe	TFSI
**C13b-C14b-C15b-C16b**	176.1(5)	177.4(4)		
**C13c-C14c-C15c-C16c**	176.2(6)	179.4(4)		
**C6-C7-C8-C9**	179.8(5)	176.5(4)	177.3(3)	171.90(14)
**C6b-C7b-C8b-C9b**	177.8(5)	179.5(4)		
**C6c-C7c-C8c-C9c**	178.9(6)	172.2(4)		

Table S12. Anion influence on cyanine chromophore geometry (°).

Atoms	torsion angles			
	O_3SMe	O_3SPh	O_3SPhMe	TFSI
**C9-C10-C11-C12**	179.5(5)	179.9(4)	180.0(3)	174.39(14)
**C9b-C10b-C11b-C12b**	178.6(5)	179.8(4)		
**C9c-C10c-C11c-C12c**	179.9(7)	176.0(4)		
**C8-N1-C1-C2**	179.6(5)	177.4(4)	179.6(4)	179.6(4)
**C8b-N1b-C1b-C2b**	179.4(5)	179.7(4)		
**C8c-N1c-C1c-C2c**	179.0(6)	172.4(4)		
**C8-C7-C6-C5**	177.5(5)	177.4(4)	178.3(4)	174.74(14)
**C8b-C7b-C6b-C5b**	178.3(5)	179.7(4)		
**C8c-C7c-C6c-C5c**	179.2(7)	174.7(4)		
**C14-N2-C21-C20**	179.5(5)	178.6(5)	179.7(4)	178.78(14)
**C14b-N2b-C21b-C20b**	176.4(5)	179.8(4)		
**C14c-N2c-C21c-C20c**	178.7(6)	178.7(5)		
**C14-C15-C16-C17**	179.9(5)	178.5(4)	179.7(4)	177.06(14)
**C14b-C15b-C16b-C17b**	178.2(6)	179.6(4)		
**C14c-C15c-C16c-C17c**	175.5(7)	179.2(5)		

Table S13. Anion charge distribution (Å).

Atoms	Anion			
	O_3SMe	O_3SPh	O_3SPhMe	TFSI
**S1-O1**	1.436(4)	1.441(10)	1.451(2)	1.4282(11)
**S1-O2**	1.462(3)	1.423(6)	1.454(3)	1.4246(11)
**S1-O3**	1.456(3)	1.519(8)	1.457(2)	
**S2-O4**	1.382(12)	1.448(4)		1.4172(12)
**S2-O5**	1.463(10)	1.447(4)		1.4259(13)
**S2-O6**	1.440(11)	1.432(4)		
**S3-O7**	1.425(14)	1.470(8)		N3-S1
**S3-O8**	1.552(10)	1.538(12)		1.5616(14)
**S3-O9**	1.502(15)	1.451(11)		N3-S2
				1.5760(14)

Table S14. Shortest anion-chromophore coordination distance (Å). The nitrogen atoms of the chromophore are acting as anchor groups for the measurement. Measurements were performed with Mercury 3.8.

Atom	Anion			
	O_3SMe	O_3SPh	O_3SPhMe	TFSI
**N1-O**	4.108	4.321	4.408	4.492
**N1b-O**	4.626	4.968		
**N1c-O**	4.254	4.342		
**N2-O**	4.244	4.619	4.129	4.424
**N2b-O**	4.266	4.807		
**N2c-O**	4.092	4.200		

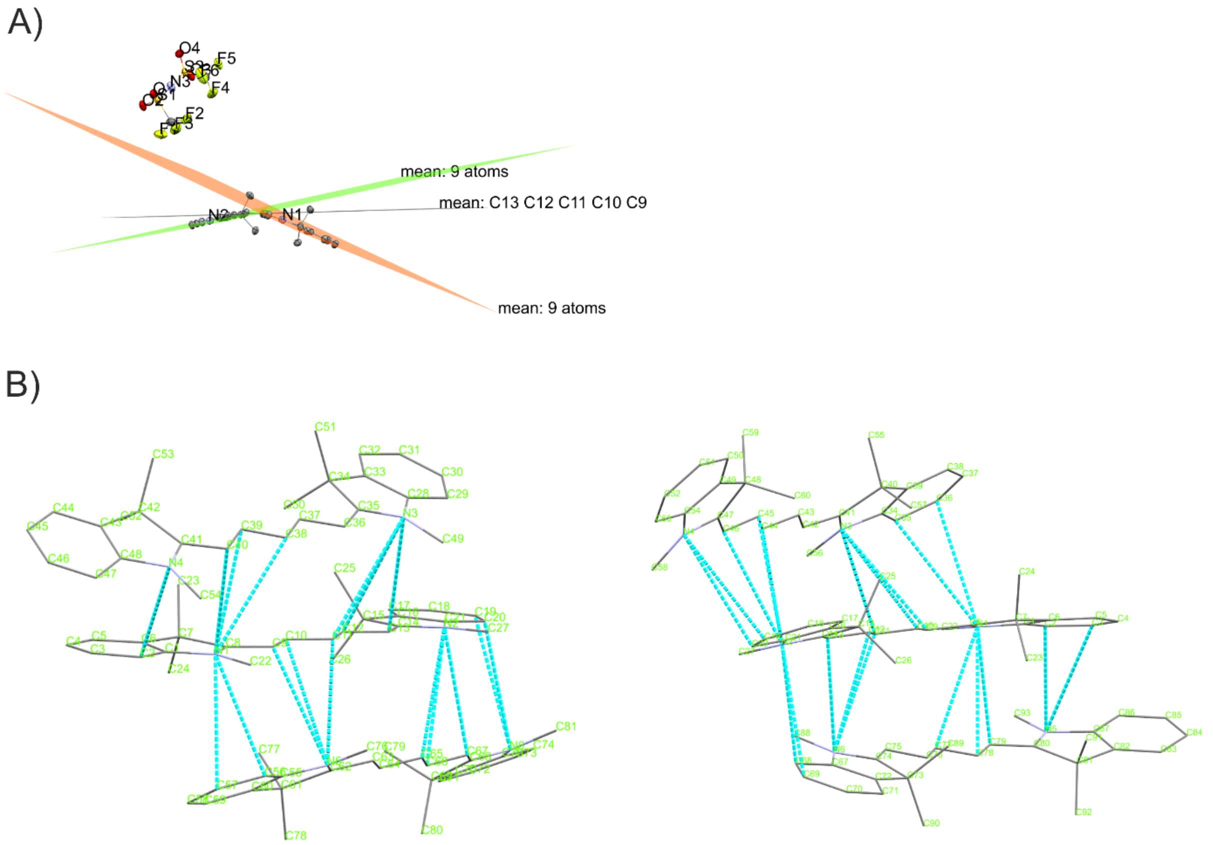

Figure S14. A) Chromophore bending angle measurement example. B) Observed π-interaction contacts and tilting within the chromophore triplets. Left: Cy5O$_3$SMe, Right: Cy5O$_3$SPh.

Organic Photovoltaic Device Fabrication

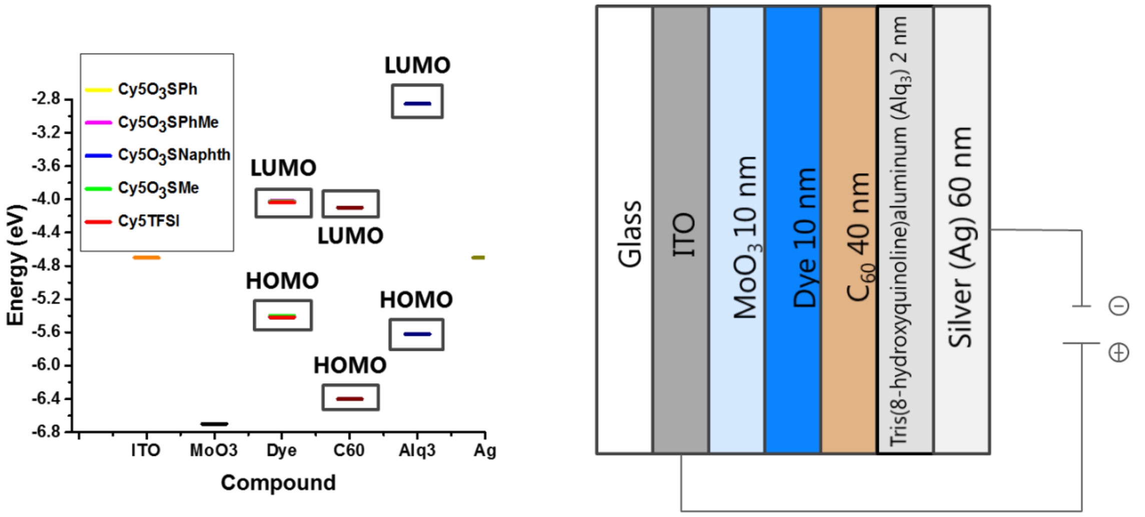

Figure S15. Band energy situation in fabricated cells against vacuum level and architecture of the manufactured cells.

The devices were made out of seven layers: glass, indium tin oxide, molybdenum oxide (10 nm) as hole transport layer, dye layer (10 nm) as light absorbing active layer, C_{60} (40 nm) as electron acceptor, aluminumquinoline (2 nm) as diffusion blocking layer and silver (60 nm) as

bottom electrode. The metals and C_{60} were deposited by thermal evaporation while the dye layer was spincast from solution.

Statistics of the Cy5TFSI Cell

- Detailed insight reveals a broad deviation in all cell parameters
- Indication that the active layer morphology is not optimal

Film surface is very smooth, which indicates that the problem lies inside the volume of the active layer

Table S15. Detailed statistics of the best performing Cy5TFSI. Only working cells were analyzed.

	N total	Mean	Standard Deviation	Minimum	Median	Maximum
**Voc (V)**	33	0.66	0.09	0.27	0.70	0.72
**Jsc (mA cm^{-2})**	33	5.82	0.50	5.00	5.79	7.37
**Eff (%)**	33	2.00	0.54	0.49	2.03	3.02
**FF (%)**	33	50.91	7.44	32.04	53.35	59.19

Optimization Trials of the Cy5TFSI Cell

Vacuum Treatment of the Active Layer

The results indicated that residues of TFP could cause broad deviations in cell performance data. Therefore after spincoating of the active layer the device was stored for 16 h at 1 x 10^{-6} mbar before further processing. All the average values improve after this additional vacuum treatment this indicates that TFP remains in the thin film volume. For further studies TFP should be avoided, even if it forms a very smooth film surface.

Table S16. Detailed statistics obtained after vacuum treatment of the active layer.

	N total	Mean	Standard Deviation	Minimum	Median	Maximum
**Voc (V)**	15	0.63	0.05	0.56	0.64	0.69
**Jsc (mA cm^{-2})**	15	5.87	0.42	5.43	5.72	6.83
**Eff (%)**	15	2.13	0.39	1.53	1.97	2.86
**FF (%)**	15	57.05	3.92	49.24	56.05	62.50

Thermal Annealing Followed After Vacuum Treatment

Remaining TFP traces could change the 3D morphology of the active layer by accelerating crystallization. Therefore thermal annealing was performed with the aim to evaporate residual TFP. Devices were heated at different temperatures for 1 h.

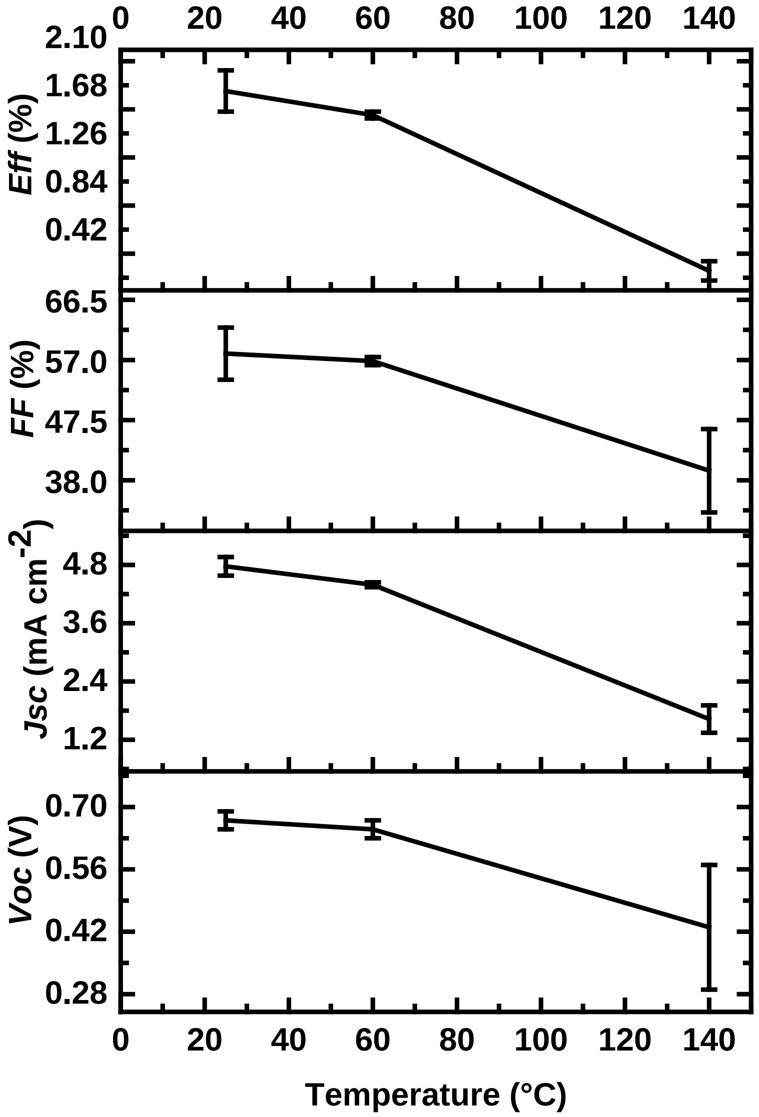

Figure S16. OPV parameters plotted against dependence of temperature. 8 Cells were investigated for each temperature step.

Unfortunately the additional thermal treatment worsens all of the OPV parameters. Optimal conditions are thus reached already after vacuum treatment.

Additive Effect

To observe if an antisolvent could prevent the TFP effect different concentrations of diiodooctane (DIO) were added to the Cy5TFSI solution before spincasting.

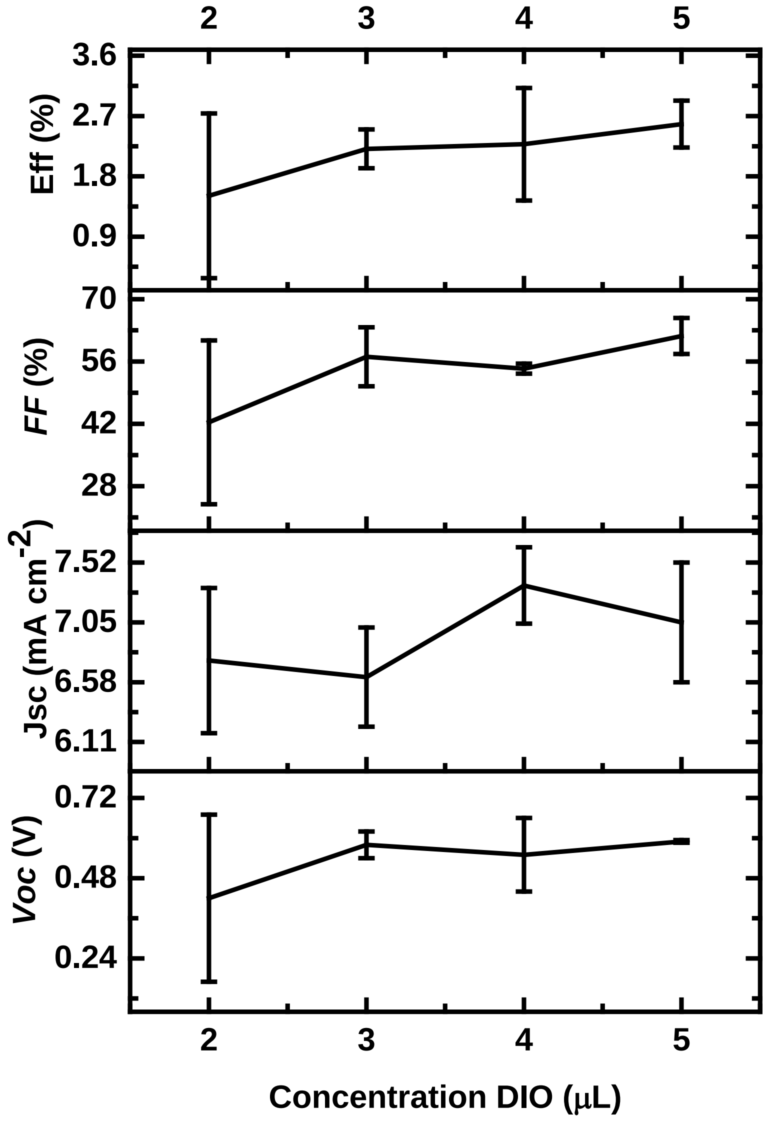

Figure S17. DIO concentration effect on OPV device parameters.

A large gap between working and short circuited cells was obtained. Only 4 of 8 cells from each device were not short circuited. A slight improvement of the fill factor and performance occurs, while the open circuit voltage and the short circuit current density remains the same. The short circuit current is increased compared to the values obtained after vacuum treatment.

All other values do not reach the averages obtained after vacuum treatment. Therefore it can be concluded that DIO does not have a significant favorable effect on device parameters.

Active Layer Thickness Variation

A thicker active layer is capable of absorbing more light and generating more free charges. Most of the organic materials have a very low exciton diffusion length of 10 nm, so that thick light absorbing layers result in charge recombination. To find out if this exciton diffusion length limitation also applies to the synthesized materials four devices with different active layer thickness were prepared.

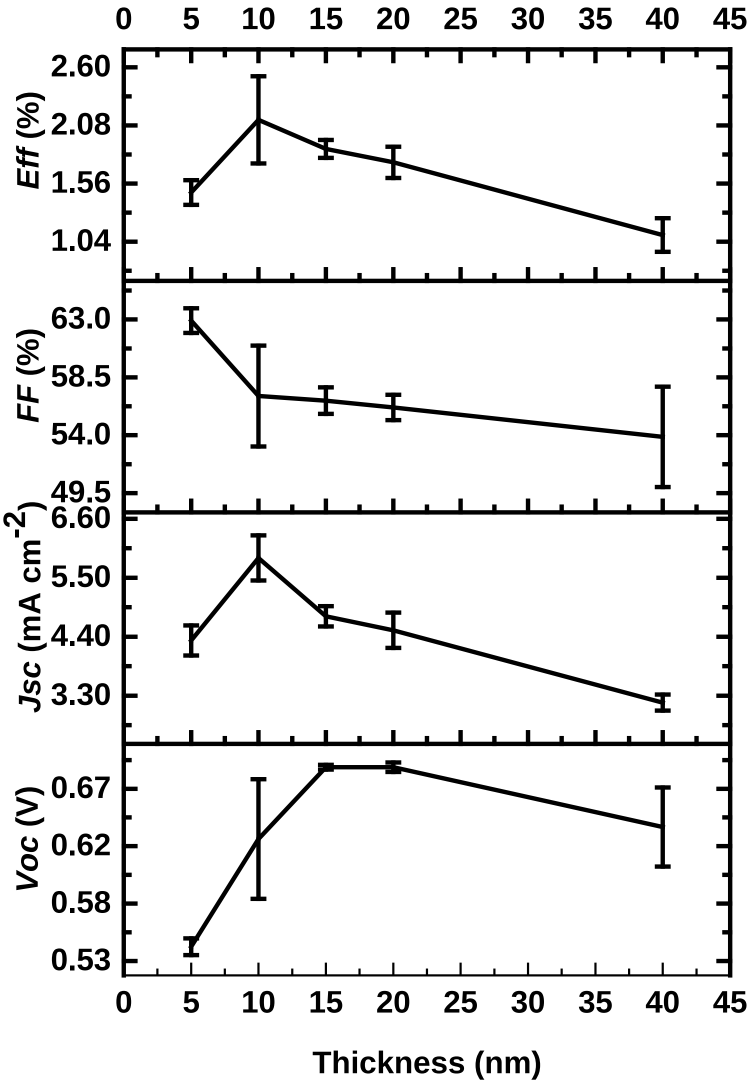

Figure S18. OPV parameters dependence on active layer thickness based on data from 8 cells for each thickness.

Except for the fill factor which reaches its maximum for an active layer thickness of 5 nm, all the OPV parameters reach their maxima at 10 nm active layer thickness. Therefore it can be assumed that the used compounds have an exciton diffusion length of 10 nm. The thickness dependent trends obtained in external quantum efficiency (EQE) spectra support this assumption.

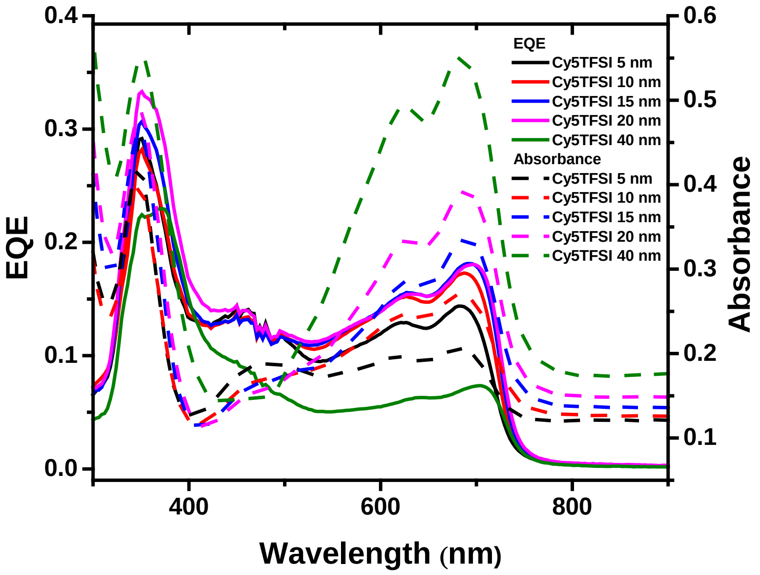

Figure S19. EQE and absorbance of aged devices (storage of several weeks in a glovebox) as function of active layer thickness.

Aging behavior

Unfortunately, devices all show a fast degradation within a few hours in the dark. Interestingly this manifests in the relative contributions of the active components to the EQE spectrum. We exemplify this behavior in bilayer solar cells using Cy5O$_3$SPh as electron donor and C_{60} as acceptor. **Figure S20** clearly shows that the cyanine contribution to the EQE decreases more significantly than the C_{60} after storing the device for 16 h in a glove box. Correspondingly, the short circuit current density $J_{sc} = \int EQE(\lambda) * \Phi_{AM1.5}(\lambda) * e \, d\lambda$ obtained by integrating over $EQE(\lambda)$, the photon flux $\Phi_{AM1.5}(\lambda)$ of the AM1.5 solar spectrum and multiplying by the elementary charge e yields values of 4.30 mA cm^{-2} and 2.94 mA cm^{-2} for fresh and aged devices, respectively.

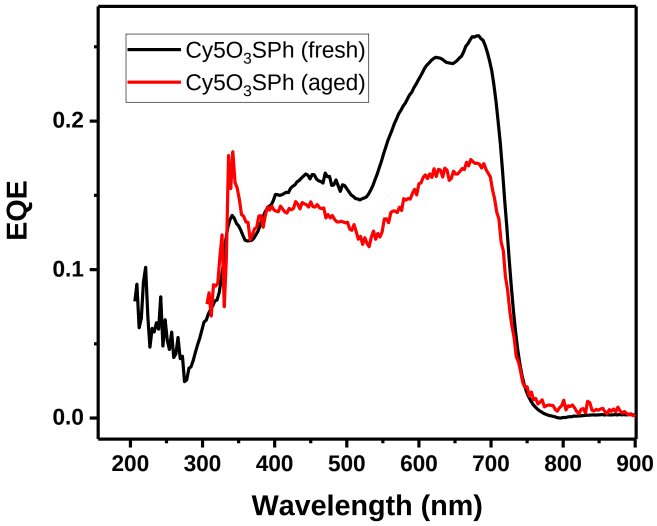

Figure S20. EQE of devices using Cy5O$_3$SPh as active layer. Fresh devices (black) were measured right after fabrication, while aged devices (red) were stored in a glovebox for 16h in the glove box.

DFT Calculations of Cyanine Dyes Based on Single Crystal Data

We have used Density Functional Theory to obtain an insight into the electronic structure and charge distribution for the different anion-chromophore pairs of Cy5O$_3$SPh, Cy5TFSI and Cy5O$_3$SPhMe and we have considered two different structures. Firstly, we have performed calculations on each inequivalent pair in the asymmetric unit cell, as obtained from the crystallographic data. Secondly, we have further energy-minimised each molecule within the unit cell at the B3LYP/6-31G* level. More specifically, all molecules within the unit cell were brought to their local minimum through an optimization within their local environment using the QM/MM scheme implemented in the NWChem software. All atoms surrounding the molecule of reference, within a radius of 12 Angstrom from the atoms in the QM part, were kept fixed, creating a neutral pocket in which the molecule was then relaxed using B3LYP/6-31G*. Since more than one molecule is present in the unit cell, this procedure was performed in a cyclic fashion, i.e. one molecule was relaxed each time in a self-consistent way until convergence was reached. Following the QM/MM optimization we computed the electronic structure for each anion-chromophore pair.

DFT calculations were performed using the Coulomb attenuated CAM-B3LYP exchange-correlation functional and a split valence double zeta polarised 6-31+G* basis set. Singlet vertical excitation energies have been computed from linear response time dependent density functional theory (TD-DFT). All calculations were performed with the NWChem program, version 6.5[5] and for the visualization of the orbitals and potential maps we used the MView software.[6]

Figure S21 displays the Kohn-Sham HOMO and LUMO orbital plots for the different anion-chromophore pairs. We find that for all compounds the HOMO and the LUMO frontier orbitals are localized on the cyanine chromophore. In particular, the LUMO displays a stronger localization over the polymethine chain compared with the HOMO and the delocalization pattern of both levels is not affected by the type of the anion. This is consistent with the cyclic voltammetry measurements suggesting that the anion does not have a strong influence on the HOMO and LUMO energy levels when considering solution or gas-phase conditions.

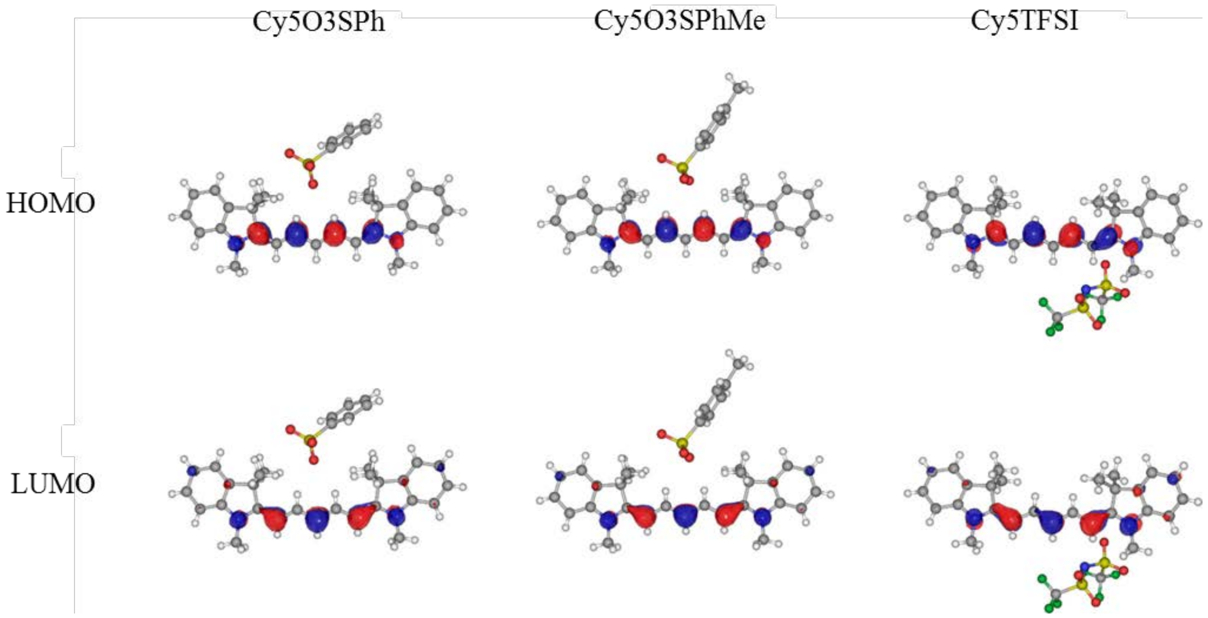

Figure S21. Kohn-Sham HOMO and LUMO frontier molecular orbital distributions of the anion-chromophore pairs calculated at the CAM-B3LYP/6-31+G* level.

The molecular electrostatic potential maps depicted in **Figure S22** can provide information about the charge distribution of each anion-chromophore pair. We observe that the negative

charge of the sulfonate anions is mainly localized on the oxygen atoms, while the bistriflylimide anion appears as a large diffuse electron cloud. The general trend is that all chromophores form an electrostatic interaction pocket in the gap between the polymethine chain and the two indolium rings. The sulfonate based anions interact electrostatically with the chromophore within this pocket. This suggests potential nucleophilic attack regions within the chromophore and represents a weak point of cyanine dye salts. Due to the crystallographic environment of the first chromophore-anion pair in Cy5O_3SPh the anion does not show strong interactions with the chromophore. A weaker electrostatic interaction with the chromophore is also observed for the bistriflylimide anion.

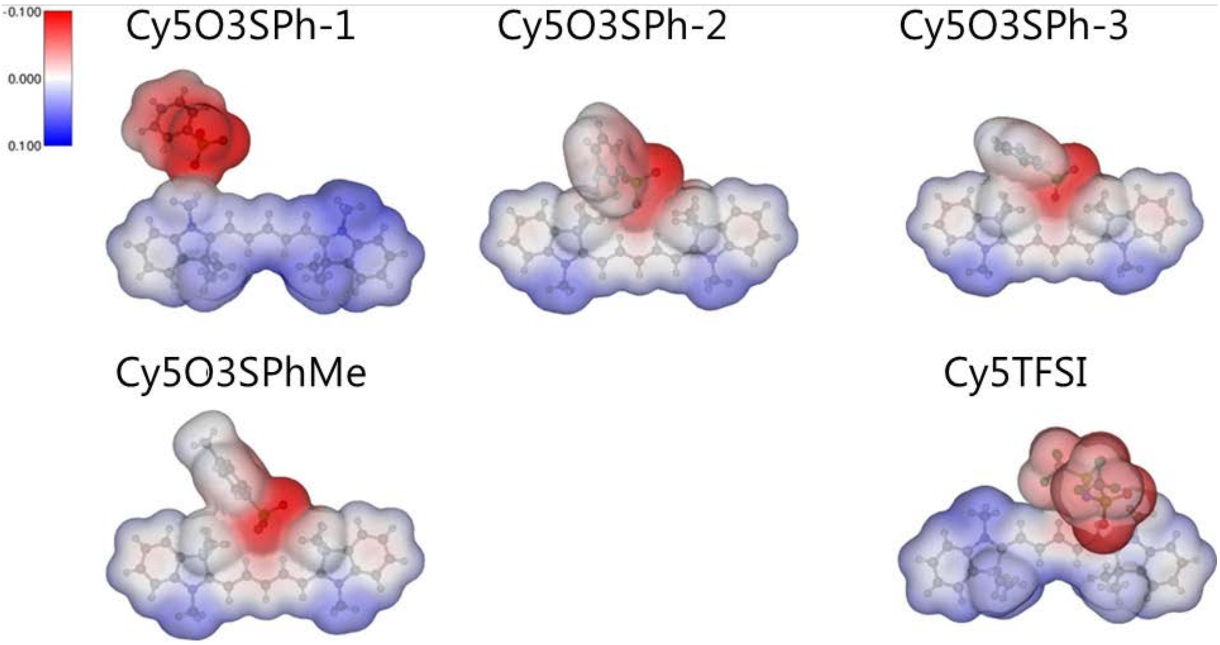

Figure S22. Molecular electrostatic potential map of the anion-chromophore pairs calculated at the CAM-B3LYP/6-31+G* level.

Literature

[1] Sheldrick, G. M. (2008). Acta Cryst. A64, 112-122.
[2] Spek, A. L. (2003). J.Appl.Cryst. 36, 7-13.
[3] C. G. Zoski, *Handbook of electrochemistry*; Elsevier, 2007.
[4] J. L. Bredas, R. Silbey, D. S. Boudreaux, R. R. Chance, *J. Am. Chem. Soc.* **1983**, *105*, 6555.
[5] M. Valiev, E. J. Bylaska, N. Govind, K. Kowalski, T. P. Straatsma, H. J. J. Van Dam, D. Wang, J. Nieplocha, E. Apra, T. L. Windus, W. A. de Jong, Comput. Phys. Commun. 2010, 181, 1477
[6] L. Viani, MView: A Tool for Visualization and Analysis of Molecular Properties, www.mview-Tools.com
